# Prognostic Factors and Survival in Pericardial Mesothelioma: A Systematic Review and Quantitative Analysis

**DOI:** 10.3390/cancers18162583

**Published:** 2026-08-11

**Authors:** Michał Kapałka, Estera Pazek, Michał Krawiec, Michał Sikorski, Grzegorz Hirnle, Tomasz Hrapkowicz

**Affiliations:** 1 Students’ Scientific Club at the Department of Cardiac, Vascular and Endovascular Surgery and Transplantology, Faculty of Medical Sciences in Zabrze, Medical University of Silesia, 41-800 Katowice, Poland; michal.kapalka00@gmail.com (M.K.); 131933@student.uni.opole.pl (M.S.); 2 Department of Cardiac, Vascular and Endovascular Surgery and Transplantology, Faculty of Medical Sciences in Zabrze, Medical University of Silesia, 41-800 Katowice, Poland; hirnle.g@gmail.com (G.H.); thrapkowicz@sum.edu.pl (T.H.)

**Keywords:** primary pericardial mesothelioma, rare heart tumor, survival

## Abstract

Background: Primary pericardial mesothelioma (PPM) is a rare and aggressive tumor. We aimed to analyze diagnostic pathways and factors influencing survival. Methods: A systematic review was conducted in accordance with PRISMA 2020 guidelines and registered in PROSPERO (CRD420261277326). MEDLINE, Web of Science, and EMBASE were searched up to March 2025. Case reports and case series published from 2010 onwards de- scribing histologically confirmed PPM were included. Data were extracted independently by multiple reviewers. Risk of bias was assessed using Joanna Briggs Institute tools. Individual patient data were pooled. Survival was analyzed using Kaplan-Meier and log-rank tests, and factors associated with survival ≥12 months were evaluated using univariable logistic regression. Results: A total of 120 patients were included (108 case reports, 4 case series). Diffuse pericardial involvement predominated (74.2%), while localized tumors occurred in 30.8%. Constrictive pericarditis was present in 31.7%. Median overall survival from diagnosis was 7.4 months (IQR 1.75–13.5). Use of CT and PET in the diagnostic process was associated with longer survival (CT: 9.1 vs. 1.5 months, *p* = 0.02; PET: 14 vs. 6 months, *p* = 0.04). Chemotherapy was associated with improved survival (OR 3.3; 95% CI 1.3–8.4; *p* = 0.01). Greater pericardial effusion volume was also associated with longer survival (OR 1.56; 95% CI 1.05–2.32; *p* = 0.028). Worse outcomes were observed in patients with constrictive pericarditis (*p* = 0.01) and diffuse disease (*p* = 0.04). Conclusions: PPM carries a poor prognosis. Chemotherapy and advanced imaging are associated with longer survival, whereas constrictive pericarditis and diffuse involvement indicate worse outcomes. Early recognition may reduce diagnostic delay. Findings should be interpreted with caution due to limitations of case-based data.

## Simple Summary

Primary pericardial mesothelioma is an exceptionally rare and aggressive cancer with a median survival of only several months. This study analyzes clinical data from the last 15 years to identify factors that improve patient outcomes. Our findings show that the use of advanced imaging, such as specialized scans, and the administration of chemotherapy are significantly associated with longer survival. In contrast, diffuse neoplastic involvement and the development of constrictive pericarditis lead to much poorer clinical results. These results are important for the medical community because they highlight the need for a high index of suspicion in patients initially suspected of having simple pericarditis. By recognizing specific predictors of survival and reducing diagnostic delays, clinicians can better tailor treatments for this challenging malignancy. This research provides a necessary update on managing a disease that remains difficult to diagnose and treat effectively.

## 1. Introduction

Primary pericardial mesothelioma (PPM) is the most common primary pericardial malignancy, though it remains a rare and aggressive disease [1]. In the Surveillance, Epidemiology, and End Results between 1973 and 2013, pericardial mesothelioma accounted for 0.4% patients diagnosed with malignant mesothelioma [2]. Autopsy data from nearly 500,000 cases indicate an incidence below 0.0022%, meaning PPM constitutes only 2% to 3% of all primary tumors of the heart and pericardium [3]. Several intrinsic features make PPM particularly difficult to diagnose. The clinical presentation is highly nonspecific: patients typically present with insidious-onset dyspnea, chest pain, cough, fatigue, weight loss, or peripheral edema, all readily attributable to far more common cardiopulmonary conditions [4,5,6]. Patients often present with pericardial effusion, signs of constrictive pericarditis, or progressive heart failure due to the tumor’s local mass effect. Because of these diagnostic difficulties, PPM is frequently discovered at an advanced stage or during autopsy, with a median survival of 6 to 10 months after detection [7]. Currently, there are no standard treatment guidelines, and clinical decisions mostly depend on published case reports rather than clinical trials. To date, only a limited number of studies have analyzed available evidence on this topic. McGee et al. evaluated survival and treatment approaches in 103 published cases, while Brydges et al. performed a population-based analysis comparing patients with pericardial and pleural mesothelioma [8,9]. Stella analyzed the clinical presentation, diagnostic workup, and administered treatments in 72 cases from the Italian National Mesothelioma Registry [10]. However, several practical gaps remain. It is still unclear which clinical features should raise early suspicion of PPM, what imaging pattern across modalities most reliably suggests pericardial malignancy, and how these elements can be integrated into a workable diagnostic pathway. Survival-associated factors, in turn, have rarely been examined with the caution that case report-based data demand. In the present study, we aimed to analyze diagnostic pathways, find out clinical red flags suggestive of PPM during the workup of pericardial disease and identify factors associated with survival in patients with pericardial mesothelioma over the past 15 years.

## 2. Materials and Methods

### 2.1. Eligibility Criteria

Case reports describing primary pericardial mesothelioma were included in the review. Studies published before 2010 were excluded, as diagnostic methods and treatment approaches at that time were not comparable to current standards. Original research articles and review papers were excluded. Case reports describing metastatic mesothelioma originating from the pleura, as well as those describing myocardial mesothelioma, were also excluded.

### 2.2. Information Sources and Search Strategy

This systematic review was conducted in accordance with the PRISMA 2020 guidelines [11]. The completed PRISMA 2020 Checklist and PRISMA 2020 for Abstracts Checklist are provided as Supplementary Files S1 and S2. The study protocol was registered in PROSPERO with ID: CRD420261277326, available at https://www.crd.york.ac.uk/PROSPERO/view/CRD420261277326, accessed: 29 May 2026. A comprehensive search of the MEDLINE, Web of Science, and EMBASE databases was conducted in March 2025. The search strategy included the following terms: (“pericardial mesothelioma” OR “primary pericardial mesothelioma” OR “malignant pericardial mesothelioma”). A publication date filter was applied during the database search to include only studies published from January 2010 onwards. The search was performed across titles, abstracts, and keywords. The initial electronic search yielded 583 records. Prior to screening, 194 duplicates were removed, and 10 records were automatically excluded as ineligible by screening tools.

### 2.3. Study Selection Process

**Figure 1:**
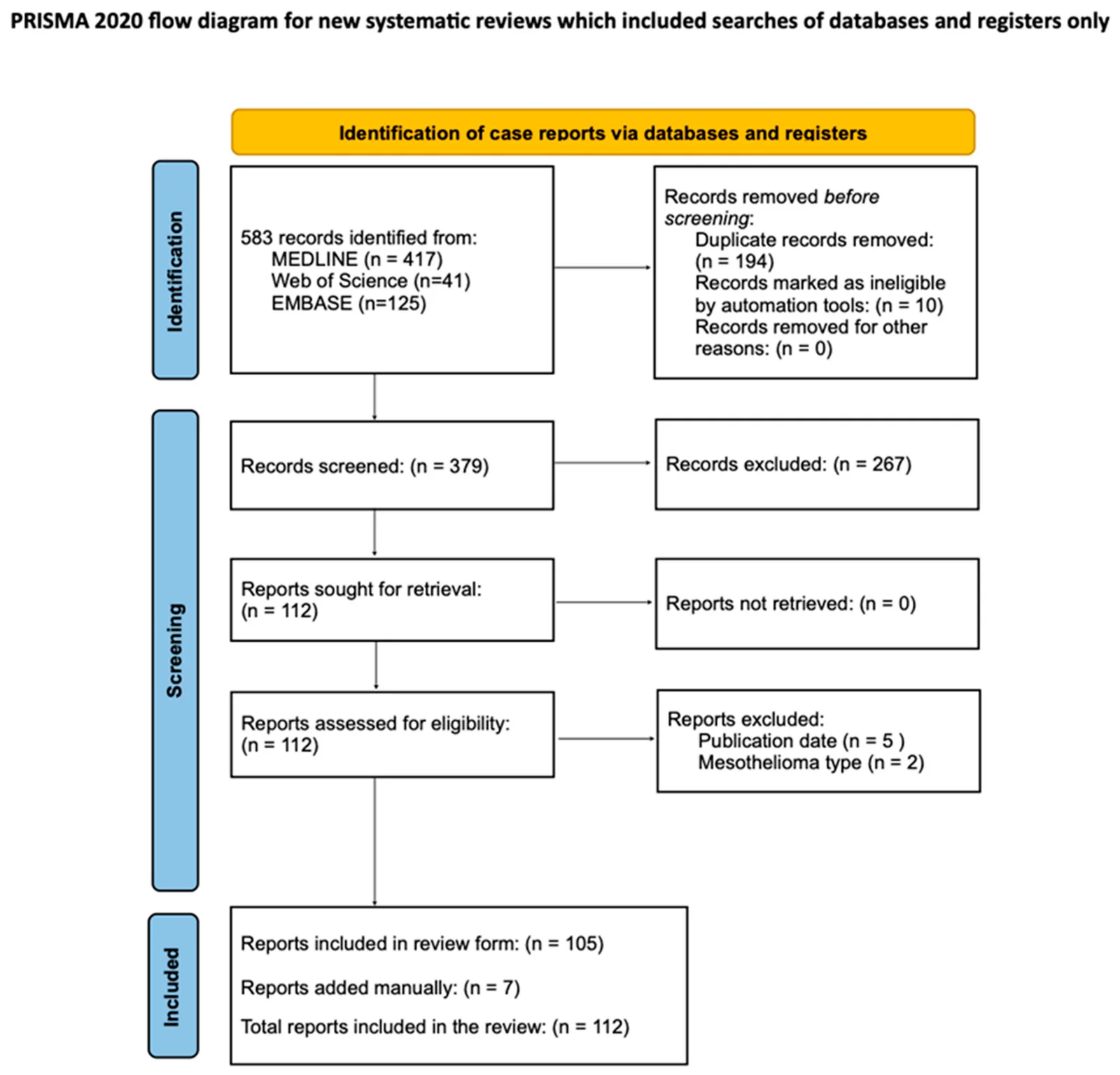
PRISMA flow diagram of the literature screening process.

The initial screening of articles was performed using Rayyan software (version 1.6.0; Cambridge, MA, USA). Two independent reviewers assessed the titles and abstracts of all retrieved studies to determine their eligibility for full-text review. Within the Rayyan platform, keyword highlighting features were used to assist manual screening: “pericardial,” “pericardium,” and “case report.” In parallel, exclusion criteria were specified to filter out studies involving “pleura,” “pleural,” “peritoneum,” “peritoneal,” and “literature review.” A total of 379 records were screened based on titles and abstracts to assess eligibility in terms of publication date, article type, origin of the neoplastic process, and exclusion of animal studies. Discrepancies were resolved through discussion and consensus. Articles that did not meet the inclusion criteria or fulfilled any of the exclusion criteria were excluded from the analysis using the Rayyan platform. A total of 112 studies were identified as potentially eligible for full-text review. Not all data points were available for each case, as not all variables were reported by the original authors. Due to the rarity of pericardial mesothelioma and the limited number of published cases, reports were not excluded based on incomplete data. A total of 105 articles identified through the database search and 7 additional articles identified through manual searching were included in the final analysis. The study selection process is illustrated in the PRISMA flow diagram (Figure 1). Detailed bibliographic characteristics of all included studies are provided in Supplementary Table S3.

### 2.4. Risk of Bias Assessment

Risk of bias was assessed independently for all included publications using the Joanna Briggs Institute (JBI) critical appraisal tools matched to study design. Single case reports were evaluated with the JBI Critical Appraisal Checklist for Case Reports, and case series with the JBI Critical Appraisal Checklist for Case Series. Each item was rated as “Yes,” “No,” “Unclear,” or “Not applicable” based on the full text of the publication. For each article, the number and proportion of “Yes” responses were calculated relative to the number of items, with “Not applicable” items excluded from the denominator. Because the JBI tools do not specify cut-off thresholds for overall risk of bias categorization, proportionally scaled thresholds were defined a priori for the purposes of this review. For case reports, low risk of bias was assigned at ≥7/8 “Yes” responses, moderate risk at 4-6/8, and high risk at ≤3/8. For case series, the corresponding thresholds were ≥8/10, 5-7/10, and ≤4/10. Across the 108 case reports, the mean score was 6.73/8 (84.2%); 75 publications (69.4%) were classified as low risk of bias and 33 (30.6%) as moderate risk, with no report meeting the criteria for high risk of bias. Among the 4 case series, the mean score was 7.75/10 (77.5%); 2 articles (50.0%) were rated as low risk of bias and 2 (50.0%) as moderate risk, with none classified as high risk. The detailed risk-of-bias assessment for all included studies is presented in Supplementary Table S4.

### 2.5. Data Collection Process

Clinical data were extracted independently by four authors from each included case report, based on the information provided by the original authors. Collected variables included patient demographics, asbestos exposure, comorbidities, tumor characteristics (localization, pericardial thickening, effusion, volume, the maximum standardized uptake value (SUVmax)), diagnostic methods, histological subtype, and presence of metastases. Details regarding misdiagnosis, initial treatment, cytology, biopsy findings, and imaging modalities (CT-computed tomography, MRI-magnetic resonance imaging, PET-positron emission tomography) were also recorded. Treatment modalities (surgery, chemotherapy, immunotherapy, radiotherapy) and survival data (overall survival, survival from symptom onset and from diagnosis, and time to diagnosis) were collected when reported.

### 2.6. Methods of Evaluating Data

Two authors searched independently for information within the included articles. Any discrepancies were resolved by consensus among all authors. Most information was directly described in the articles. Some information was not precisely specified and requires careful assessment. In cases where pericardial effusion amounts were described in words, they were classified as minimal distance in millimeters along with ESC guidelines: 2 mm in mild, 10 mm in moderate and 20 mm in large effusion. If the term biopsy was not specified as surgical, it was classified as a percutaneous biopsy. Positive cytology results were defined as atypical mesothelial cells in pericardial fluid. Initial treatment for idiopathic pericarditis consisted of steroid therapy and colchicine without defining etiology of pericarditis. Treatment for different etiology indicates settling diagnosis of, e.g., pericarditis in the course of tuberculosis or systemic rheumatoid disease. Pericardial mesothelioma was classified as diffuse when described as extensive, circumferential involvement of the pericardium, typically presenting as relatively uniform pericardial thickening encasing the heart. Localized mesothelioma was defined as a focal or well-circumscribed lesion limited to a specific region of the pericardium. Classification was based on morphological descriptions provided in the original reports derived from CT, MRI, echocardiography, surgical exploration, or autopsy. We acknowledge, however, that no universally accepted radiological or pathological criteria currently exist for this distinction in PPM.

### 2.7. Statistical Analysis

Statistical analysis was performed using Statistica software (TIBCO Software Inc., Palo Alto, CA, USA). Results were presented using descriptive tables and Kaplan-Meier survival curves. For descriptive analyses, all 112 included publications were eligible regardless of data completeness. For all further analyses, only cases with available data for a given variable were included in each analysis. No data imputation was performed. For survival analyses and logistic regression, only cases with a known follow-up duration and a defined outcome at the end of follow-up (death or alive) were included. Patients diagnosed at autopsy were excluded from survival analyses based on time from diagnosis, as no measurable post-diagnosis survival time was available. Due to heterogeneity and incomplete reporting of follow-up duration across case reports, logistic regression was used instead of time-to-event multivariable analysis. The Shapiro-Wilk test was used to assess the normality of variable distributions. Two survival endpoints were analyzed: (1) overall survival from symptom onset to death or last follow-up, and (2) overall survival from diagnosis to death or last follow-up. Patients alive at the time of the last follow-up or lost to observation were censored at the date of their last documented clinical contact. Survival analyses were performed separately for each endpoint. The Mann-Whitney U test was applied to compare the duration of the diagnostic process and survival times between patient subgroups. For comparisons involving more than two groups, the Kruskal-Wallis test was used. Univariable logistic regression was performed for each survival endpoint to identify factors associated with survival beyond ≥12 months. Survival analyses between subgroups were performed separately for each endpoint using the Kaplan-Meier method, and differences between subgroups were evaluated using the log-rank test. Only cases with available data for a given variable were included in each analysis. Results of logistic regression were presented as odds ratios with 95% confidence intervals (CIs). A *p*-value < 0.05 was considered statistically significant.

## 3. Results

### 3.1. Baseline: Case Characteristics, Exposures, and Clinical Presentation

A total of 120 cases of PPM were identified between 2010 and 2025, comprising 4 case series and 108 case reports. The median age at diagnosis was 54 years (range: 17–85 years; interquartile range [IQR]: 42–66 years). The demographic characteristics of the analyzed cohort are presented in Table 1.

In the analyzed group, there were 55.8% men and 44.2% women. Suspected or confirmed asbestos exposure was reported in 11.67% of patients; however, only 71 authors specified whether such exposure had occurred. Histological subtyping was available for 88 cases, with the details provided in Table 2. The epithelioid subtype was the most prevalent, accounting for 67.05% of cases, while the sarcomatoid and biphasic subtypes were less common, accounting for 11.36% and 17.05% of cases, respectively. Other subtypes were reported in 4.55% of cases. In 26.6% of patients, the histological subtype was not determined. Among these cases, pre-existing conditions were prevalent, with cardiovascular comorbidities accounting for 20%, other comorbidities for 21.7%, and prior malignancies for 13.3%. Pericardial effusion occurred in 75.8% of cases, representing the most frequent imaging finding, whereas features consistent with constrictive pericarditis were documented in 31.7% of patients. The median pericardial thickness assessed in imaging was 19 mm (IQR: 13.8–20 mm). There were two types of mesothelioma neoplastic process distinguished in the cohort. Localized processes described also as pericardial tumors were identified in 30.8% (*n* = 37) of cases and diffuse processes in 74.2% (*n* = 89) defined as even pericardial thickening with features of chronic inflammation; 5% of patients presented both types concurrently. Metastases were observed in 18.3% of cases, consisting of locoregional spread within the mediastinum, lymph node involvement in 10.8%, and distant metastases in 13.5%. In 5% of patients, both local and distant metastases were present.

As pericardial mesothelioma is known for difficulties in the diagnostic process, there were assessed factors affecting the time of the diagnostics Table 3. Constrictive pericarditis was a clinical parameter strongly associated with delayed diagnosis. Misdiagnosis, defined as diagnosis of idiopathic or specific type pericarditis (other than malignant) etiology in the initial phase, prolonged the time to diagnosis to 6.5 months. In patients with constrictive pericarditis, the median diagnostic delay was 8 months compared with 3 months in those without this condition. Patients presenting a diffuse neoplastic process experienced a longer median time to diagnosis (5.5 vs. 2 months).

### 3.2. Diagnostic Approach

The method of diagnosis was outlined in 118 cases, as detailed in Table 4. Chest CT results were available in 77% of cases, MRI in 28.2%, and PET scans in 44.2% of cases. In the subgroup of patients (*n* = 17) who underwent PET scanning, SUVmax had a median of 9.2, with an IQR of 6.7 to 17.8. The use of PET in the diagnostic process deserves particular attention. Incorporating PET into the diagnostic pathway was notably correlated with longer survival, with a median of 14 months compared to 6 months without it. Utilization of CT imaging at diagnosis was also linked to a markedly improved median survival (9.1 months) compared with patients who did not undergo CT imaging (1.5 months). These data confirm the importance of accurate imaging in optimizing treatment.

Pericardial fluid cytology was performed in 58 patients (48.3%) and was positive in 22 cases (37.9%). Overall, 93 patients (77.5%) underwent at least one biopsy procedure. Surgical biopsy was the most common approach, performed in 76 patients (63.3%), while endoscopic (VATS or endobronchial) and percutaneous biopsies were used in 9 (7.5%) and 8 patients (6.6%), respectively. Definitive diagnosis was most frequently established through surgical biopsy or during pericardiectomy, accounting for 68.3% of cases (*n* = 82). In contrast, non-surgical biopsy techniques provided the diagnosis in 20.0% of patients (*n* = 24), whereas pericardial fluid cytology alone was diagnostic in only four cases (3.3%). The diagnostic interval was longer among patients who underwent thoracoscopic biopsy, with a median time to diagnosis of 6 months compared with 3 months in those who did not undergo this procedure. Post-mortem diagnosis was made in eight patients (6.7%). Initial biopsy results were inconclusive in 15 cases (12.5%). In three of these patients, fluorescence in situ hybridization (FISH) was subsequently used to confirm the diagnosis. Following an inconclusive non-surgical biopsy, six patients required surgical biopsy, while another six underwent a repeat surgical biopsy.

Although cytology of pericardial fluid was performed in 58 cases (48.3%), a positive cytological result, revealing the presence of malignant cells, was obtained in 36 cases (46.8%). In 3.3% cases, the diagnosis was based solely on a positive cytological examination of pericardial fluid. Seventeen authors reported positive biomarkers in pericardial fluid. There were eight patients with positive hyaluronic acid, six with positive lactate dehydrogenase, three with positive adenosine deaminase, four with carcinoembryonic antigen and two with squamous cell carcinoma antigen. In 85 cases, the diagnostic method raised oncological suspicion. CT was the most frequently used tool, leading to a suspicion of malignancy in 38 patients. Ultrasonography was another common method, identifying suspicious lesions in 30 patients. PET led to a suspicion of cancer in nine patients, while MRI did so in six cases. X-ray imaging was the least likely to raise oncological suspicion, doing so in only two cases. Initial misdiagnosis was reported in 34.2% of cases, being incorrectly attributed to tuberculous pericarditis.

### 3.3. Treatment and Clinical Outcomes

Treatment data is provided in Table 5. A total of 84 patients underwent any causal treatment. The eight patients diagnosed post-mortem were excluded from the outcome analysis. Therapeutic surgical treatment was performed in 78 patients; however, owing to repeated interventions in some individuals, the cumulative number of surgical procedures reached 85. Overall, seven patients (5.8%) underwent more than one surgical procedure. Debulking surgery was the most frequently performed intervention, accounting for 29 procedures (34.1%), closely followed by pericardiectomy for constrictive physiology (32.9%, *n* = 28). Window or relief procedures represented 23.5% of interventions (*n* = 20), whereas curative or localized tumor resection was achieved in only eight cases (9.4%). Radiation therapy was administered in 10 cases (8,3%), while surgical intervention, including pericardiectomy and/or mass resection, was performed in 52.5% of patients. The absence of surgical intervention was associated with a shorter survival time from diagnosis-the median was 3 months, compared to 8 months in patients who underwent surgical treatment. Information regarding chemotherapy was available for 46 cases (38.3%).

The most pronounced survival benefit was observed in patients who received chemotherapy. The administration of chemotherapy conferred a survival benefit relative to patients who did not undergo systemic treatment. Patients receiving chemotherapy had a median survival from symptom onset that was approximately four times longer than that of untreated patients (16 months vs. 4.75 months). Moreover, a similar pattern was observed for median survival from diagnosis, which was 3 months in patients without chemotherapy, in contrast to 9 months in those who underwent systemic treatment. Stratification by exact line of therapy (first-line vs. subsequent lines) and treatment intent (adjuvant vs. palliative) was not possible due to a lack of reporting in the primary literature. Among the 46 patients with available chemotherapy data, 4 received single-modality therapy, 28 were treated with bimodality therapy, and 14 patients underwent trimodality therapy. In the group of 47 patients who received pharmacological treatment, the predominant regimen was a combination of pemetrexed and a platinum compound, used in 55.3% of cases. Combination therapy with gemcitabine was applied in 14.9% of patients. Both the combination with bevacizumab and other multi-drug regimens were used with equal frequency, each in 8.5% of cases. Immunotherapy was implemented in 13.04% of patients as part of the systemic treatment protocols.

### 3.4. Overall Survival and Associated Factors

Of the 120 patients identified from the literature, 8 were diagnosed at autopsy and were excluded from time-to-event analyses, leaving 112 patients diagnosed pre-mortem. Of these, 42 lacked sufficient information for survival analysis from the time of diagnosis (missing follow-up duration and/or vital status) and were also excluded. The final analytic cohort therefore comprised 70 patients (62.5% of the pre-mortem subset), of whom 49 (70.0%) died during follow-up and 21 (30.0%) were alive at last reported follow-up and were right-censored. Survival outcomes are summarized in Table 6.

The median survival time from symptom onset to death was 9 months (IQR = 2.5–17), while the median survival time from diagnosis to death was 7 months (IQR = 1.5–12). Additionally, the median diagnostic interval from first symptoms to diagnosis was 4 months (IQR = 1–11).

In the correlation analysis of factors affecting survival from symptom onset in mesothelioma, both detrimental and favorable variables were identified. Cardiovascular comorbidities and a prior history of malignancy were linked to markedly shorter survival, with median values 4 and 12 times shorter, respectively, than in patients without these conditions. Patients who initially were misdiagnosed and received treatment for idiopathic or pericarditis of different etiology had a significantly longer median survival (14.25 vs. 5 months, *p* = 0.008). The use of PET (*p* = 0.04) or CT (*p* = 0.02) in the diagnostic process was also associated with longer survival (median 14 vs. 6 months and 9.125 vs. 1.5 months, respectively). Moreover, patients who received chemotherapy had significantly longer survival compared to those who did not (16 vs. 4.75 months, *p* = 0.0006) (Table 7).

Among censored patients, the median follow-up was 12 months (IQR 9–24; range 1.5–86 months). The median follow-up calculated by the reverse Kaplan-Meier method was 24 months. The distribution of follow-up duration among censored patients was as follows: <6 months, *n* = 2 (10%); 6–12 months, *n* = 8 (38%); 12–24 months, *n* = 5 (24%); ≥24 months, *n* = 6 (29%). Factors associated with survival from diagnosis are summarized in Table 8. Female sex was associated with longer survival compared to male patients (*p* = 0.04). The presence of pericardial effusion was also linked to longer survival (*p* = 0.04). A trend toward longer survival was observed among patients who underwent surgical treatment (*p* = 0.06). Finally, chemotherapy was significantly associated with prolonged survival (*p* < 0.001).

**Figure 2:**
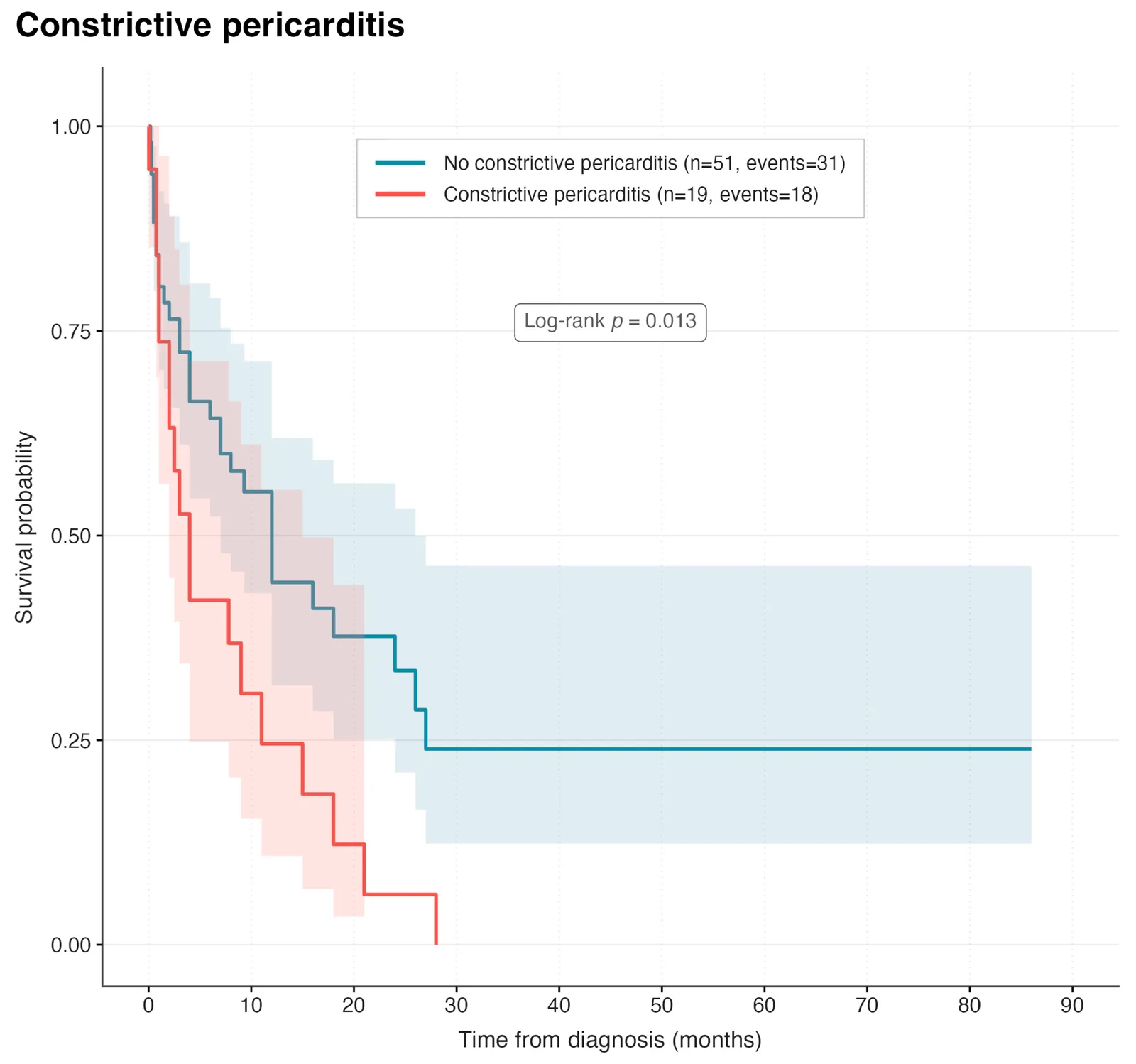
Kaplan-Meier survival curve comparing patients with constrictive pericarditis and without. Shaded areas represent the 95% confidence intervals for the Kaplan-Meier survival estimates.

**Figure 3:**
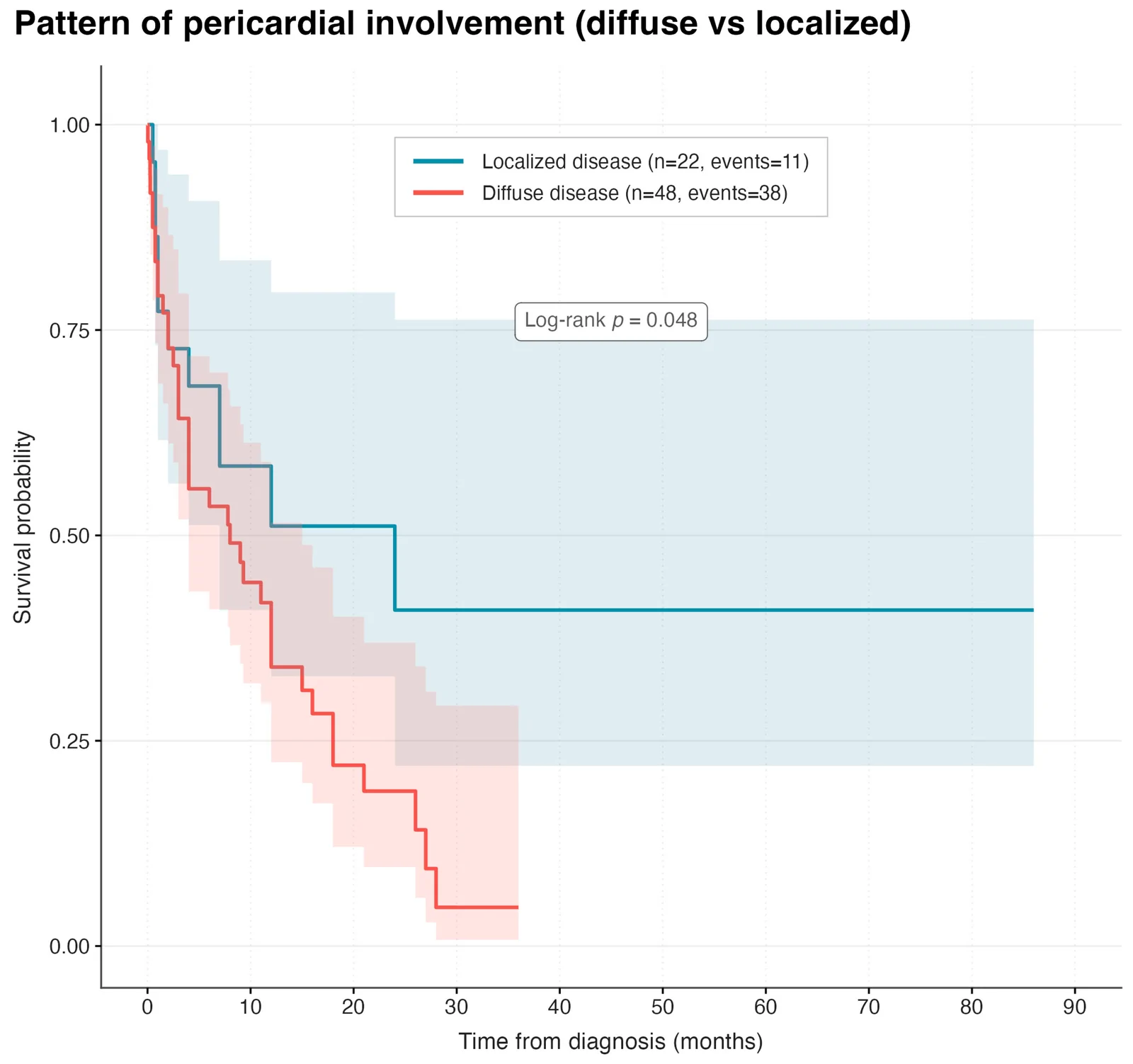
Kaplan-Meier survival curve comparing patients with diffuse and localized pericardial mesothelioma. Shaded areas represent the 95% confidence intervals for the Kaplan-Meier survival estimates.

Kaplan-Meier survival analysis from diagnosis with log-rank testing identified several factors associated with differences in survival. Patients with constrictive pericarditis (*p* = 0.019) and diffuse pericardial involvement (*p* = 0.04) had significantly lower survival rate, while use of chemotherapy was significantly associated with higher survival rate (*p* = 0.0008) (Figure 2 and Figure 3). Other subgroup analyses were performed but did not reach statistical significance. No significant association was found between age and survival from the time of diagnosis. Complete Kaplan-Meier survival analysis results are presented in Supplementary Tables S5 and S6. Survival analysis from the onset of symptoms showed longer survival in patients without cardiovascular comorbidities (*p* = 0.01), treated as other specific type pericarditis (0.003) and in those receiving chemotherapy (*p* = 0.0003). Kaplan-Meier curves for surgical treatment and chemotherapy are presented in Supplementary Figures S1 and S2. No significant differences in survival were observed across histological subtypes or treatment modalities. A meaningful negative correlation was observed between age and survival from symptom onset, indicating shorter survival with increasing age. 

### 3.5. Logistic Regression Analysis

In univariable logistic regression, advanced age was also a positive predictor for longer survival from symptoms onset [OR = 1.037; CI 1.006–1.069; *p* = 0.01]. Patients with survival ≥12 months had median 59 years compared to median 50 years with death during the first year of follow-up. The amount of pericardial fluid was associated with survival ≥12 months from symptom onset [OR = 1.56; CI:1.05–2.32 *p* = 0.028]; it was associated with negative correlation between effusion in millimeters and time of diagnosis. Misdiagnosis was associated with a higher likelihood of survival ≥12 months from symptom onset [OR = 3.37; 95% CI: 1.23–9.22; *p* = 0.018]. Similarly, the use of MRI in the diagnostic process was associated with longer survival [OR = 2.85; 95% CI: 1.04–7.83; *p* = 0.042]. These results were attributable to time of the diagnostics process. Univariable logistic regression of survival from the diagnosis revealed chemotherapy [OR = 3.3; CI 1.3–8.4; *p* = 0.01] and positive result of pericardial fluid cytology test [OR 7.1; CI 1.7–28.9; *p* = 0.007] as predictors of longer survival. Complete logistic regression results are presented in Supplementary Tables S7 and S8.

### 3.6. Cox Regression

Univariable Cox regression (Table 9) identified chemotherapy as the strongest predictor of survival (HR 0.37, 95% CI 0.20–0.66; *p* < 0.001) and showed a borderline association with diffuse pericardial involvement (HR 1.94, 95% CI 0.99–3.82; *p* = 0.055). Other variables-age ≥ 60 years, male sex, histological subtype, and surgery-did not show statistically significant associations with overall survival in univariable analysis.

In the multivariable Cox model adjusted for age, sex, histological subtype, diffuse pericardial involvement, surgery, and chemotherapy (Table 9), diffuse pericardial involvement and chemotherapy emerged as independent predictors of survival. Patients with diffuse disease had a significantly higher risk of death (aHR 2.08, 95% CI 1.02–4.25; *p* = 0.045), whereas chemotherapy was associated with a significantly reduced mortality risk (aHR 0.40, 95% CI 0.21–0.77; *p* = 0.006). Age ≥ 60 years, male sex, non-epithelioid histology, and surgery were not significantly associated with survival in the adjusted analysis (all *p* > 0.05). Detailed Cox regression results are presented in Supplementary Tables S9–S11.

## 4. Discussion

### 4.1. Epidemiology

PPM is an exceptionally rare malignancy. It represents less than 1% of all mesothelioma cases, accounting for approximately 0.7% of tumors arising at any anatomical site [8,12,13]. Its estimated frequency in unselected autopsy series ranges between 0.002 and 0.006%, corresponding to approximately 0.36 cases per 10 million person-years [12,14]. In comparison, pleural mesothelioma accounts for the majority of cases (70–80%), followed by the peritoneal subtype (20–25%) [8,15]. Globally, only a few hundred PPM cases have ever been documented in the literature [16]. Despite this rarity, PPM remains the most frequent primary neoplasm of the pericardium—an otherwise uncommon site of oncogenesis [17]. By contrast, secondary involvement of the pericardium due to metastatic spread from lung, breast, or hematologic malignancies is considerably more prevalent [17]. Most patients present in mid-adulthood (median age 50–55 years) but cases have ranged from the teenage years to the elderly [8,18]. A slight male predominance has been described in several series; however, some studies have noted a relatively higher proportion of female patients compared with pleural mesothelioma [9,19]. PPM has been reported worldwide, with case series from North America, Europe, and Asia (including a Chinese series of 164 collected cases), demonstrating that, while extremely infrequent, this cancer is a global phenomenon [14,20]. Unlike pleural mesothelioma, the etiologic link to asbestos exposure in pericardial mesothelioma remains uncertain. No definitive occupational exposure history is identified in the majority of PPM patients [12]. In the Italian registry study, only 15 of 58 patients (26%) had documented asbestos exposure [14]. Trends in PPM incidence do not parallel pleural mesothelioma (which rose and fell with industrial asbestos use), and notably, no pericardial mesotheliomas were seen in some large cohorts of heavily asbestos-exposed workers [6,21]. There have been rare reports of PPM arising after high-dose mediastinal radiation. Notably, pericardial mesotheliomas occurring years after mantle-field irradiation for Hodgkin lymphoma have been described [15]. Chronic irritation and long-standing pericarditis have also been proposed as possible contributors in selected patients, but the available data remain sparse [14]. In most cases, PPM is essentially idiopathic with no identifiable external carcinogen.

The exact mechanisms driving PPM are not as well-characterized as those for pleural diseases [22]. Even without large-scale genomic data or a clearly identified tumor-specific oncogenic driver, we know that PPM generally shares its molecular landscape with other mesotheliomas, meaning its pathogenesis is heavily rooted in tumor suppressor loss [23]. In practice, this makes evaluating BAP1 expression loss via immunohistochemistry (IHC) and checking for homozygous CDKN2A (p16) deletions via FISH invaluable [24]. Both techniques provide the high specificity required to separate malignant mesothelial cells from benign reactive proliferations, which often appear morphologically identical and cannot be reliably distinguished via conventional fluid cytology alone [25,26]. Although tissue biopsy is required, small pericardial specimens present significant challenges, including tissue fragmentation, crushing artifacts, fixation issues, and the difficulty of confirming true stromal invasion on superficial samples [27]. Ultimately, the most reliable way to optimize diagnostic yield and reach a definitive confirmation is to integrate ancillary BAP1 IHC and p16 FISH testing directly into the initial biopsy protocol [24].

### 4.2. Clinical Features

PPM typically presents insidiously and at an advanced stage, often with nonspecific cardiopulmonary symptoms that lead to initial misdiagnosis [3]. One analysis found the interval from first presentation to correct diagnosis exceeded 6 months in many cases [12]. The initial manifestations most commonly stem from pericardial effusion, with patients experiencing progressive dyspnea, orthopnea, and chest discomfort [24]. As the disease advances, additional respiratory symptoms such as cough, shallow breathing, or paradoxical pulse may develop, often accompanied by systemic features including fever, night sweats, and generalized fatigue [3]. Beyond these signs, patients may present with complications ranging from cardiac tamponade to constrictive pericarditis and heart failure secondary to tumor invasion of the myocardium [28]. These manifestations frequently mimic more common conditions, such as coronary artery disease, cardiomyopathy, tuberculous pericarditis, melanoma, hematologic malignancies, or secondary pericardial involvement from metastatic tumors [29,30].

### 4.3. Diagnostics Process: Imaging, Red Flags and Potential Diagnostic Pathway

#### 4.3.1. Histopathological Diagnosis and Cytological Assessment

Pericardial mesothelioma is histologically classified into epithelioid, sarcomatoid, and biphasic subtypes, with the epithelioid form associated with a more favorable prognosis [31]. Morphologic assessment combined with immunohistochemical positivity for mesothelial markers (calretinin, CK5/6) and negativity for adenocarcinoma markers (CEA, TTF-1) enables accurate differentiation from reactive mesothelial proliferations and other neoplasms [30,32,33]. Initial assessment of the pericardial fluid was based on echocardiographic imaging and, if pericardiocentesis was performed, cytological analysis. In systematic review, primary cardiac tumors represent 24% causes of malignant pericardial effusion [34]. According to the guidelines, cytological analyses of pericardial fluid are mandatory for the confirmation of malignant pericardial disease. Saab reported only three false negative results in cytology evaluating all pericardial effusions [35]. Takeda’s study confirmed significantly higher sensitivity of pericardial fluid cytology compared to biopsy (84.8% vs. 65.7%) in patients with malignant effusions [36]. In our analysis, 36 patients (62%) had false negative cytology results, which precludes the use of cytological examination as a reliable tool for ruling out a diagnosis of mesothelioma. On the other hand, patients with a positive cytological result exhibited longer survival from diagnosis, which closely mirrors a significantly shorter time to establish the definitive diagnosis in this subgroup (6 vs. 2.65 months). Rather than acting as an independent biological prognostic factor, a positive cytological assessment appears to accelerate the diagnostic timeline, thereby minimizing the loss of therapeutic opportunity. Therefore, in cases of negative pericardial fluid cytology, clinicians should not dismiss the suspicion of malignancy. The final diagnosis was established based solely on cytological examination in four cases, while the remaining patients required an additional pericardial biopsy to confirm the diagnosis; however, cytology at least accelerated the diagnostic process.

#### 4.3.2. Imaging Modalities in PPM: Literature Evidence

**Figure 4:**
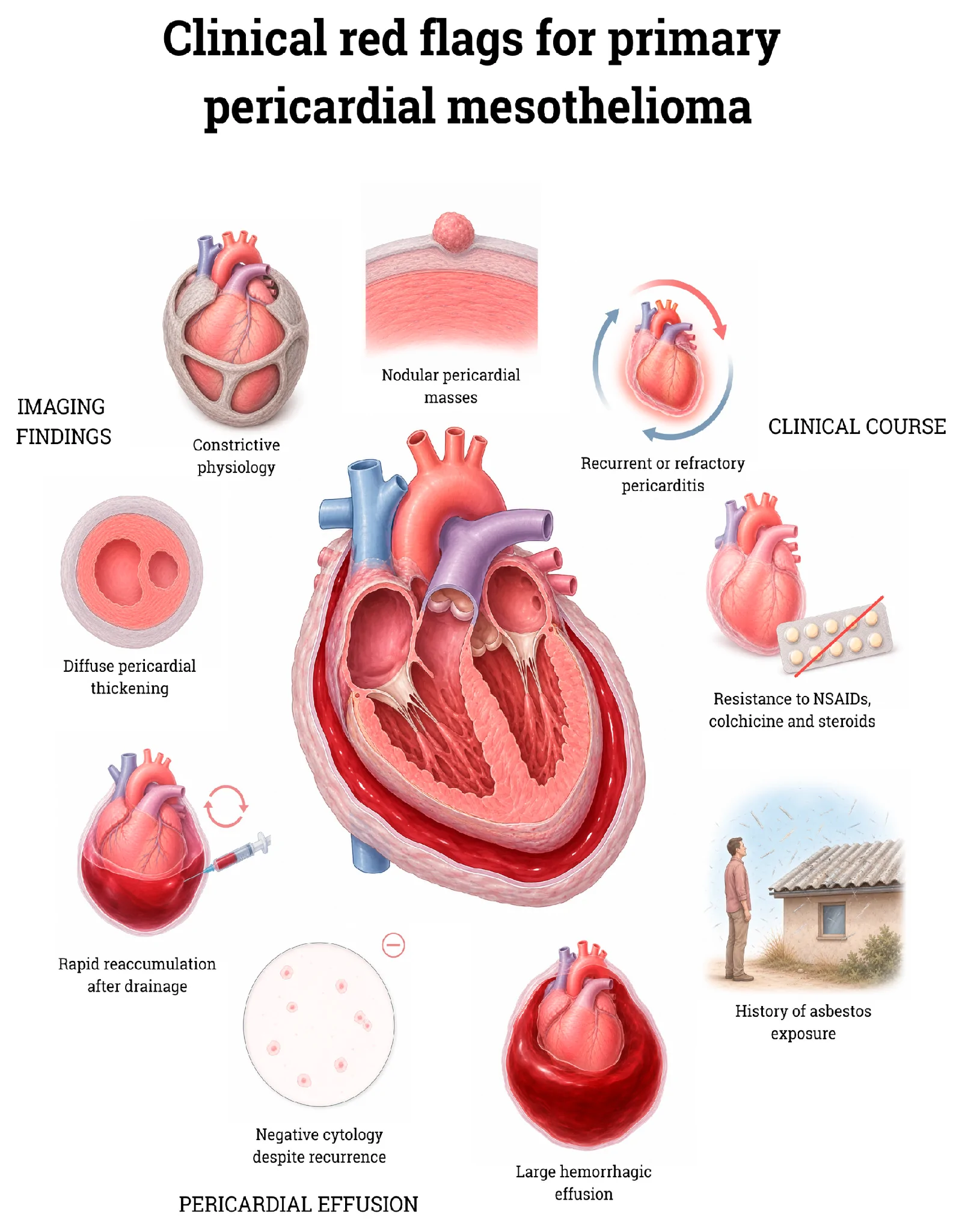
Clinical red flags for primary pericardial mesothelioma (PPM). PPM may be hidden among patients presenting with refractory or recurrent pericarditis, and its diagnosis is frequently delayed. The figure summarizes clinical features that should raise suspicion of PPM, grouped into three complementary domains: clinical course (recurrent or refractory pericarditis, resistance to NSAIDs, colchicine and steroids, history of asbestos exposure), imaging findings (diffuse pericardial thickening, constrictive physiology, nodular pericardial masses), and pericardial effusion characteristics (large hemorrhagic effusion, rapid reaccumulation after drainage, negative cytology despite recurrence). None of these features is pathognomonic in isolation; however, their co-occurrence-particularly across more than one domain—should prompt consideration of PPM and trigger advanced diagnostic workup (see Figure 5).

Echocardiography remains the primary method to assess effusion volume, tamponade physiology, and constriction [37]. It is more important while in the analysis 75.8% patients presented pericardial effusion and 31.7% had constrictive pericarditis diagnosed. According to Kong et al., pericardial effusion was the most common finding (85.9%), with the majority being massive (67.2%). Among the cases describing fluid characteristics, 95.4% were bloody. Pericardial wall thickening and pericardial masses were reported in 18.8% and 32.8% of patients, respectively. Non-echocardiographic imaging modalities detected wall thickening significantly more frequently than echocardiography (63.5% vs. 17.3%) [38]. Building on these observations, we propose a practical bedside framework of clinical red flags for PPM, grouped into three complementary domains—clinical course (recurrent or refractory pericarditis, resistance to NSAIDs, colchicine and steroids, history of asbestos exposure), imaging findings (diffuse pericardial thickening, constrictive physiology, nodular pericardial masses), and pericardial effusion characteristics (large hemorrhagic effusion, rapid reaccumulation after drainage, negative cytology despite recurrence)—that should raise suspicion of PPM and prompt further diagnostic evaluation (Figure 4) [4,6,38,39]. Among patients with constrictive pericarditis in CT and ultrasound (US) pericardium had correspondingly 6.21 mm and 4.42 mm compared to 0.6 mm and 1.2 mm in the control group [39]. In the analyzed population, the median pericardial thickening in the diffuse form was 19 mm, which underscores that any noticeable pericardial thickening should raise oncological suspicion and prompt further evaluation for possible malignant involvement. In the diagnostic process, contrast-enhanced echocardiography can be helpful. Wang underscored its diagnostic value, for it helped visualize hyperenhanced pericardial mass with a radial enhancement pattern in which there were multiple blood vessels [40]. Wang’s study showed that contrast-enhanced echocardiography has a high diagnostic accuracy for the differentiation of a benign tumor from a malignant tumor [40].

PET imaging can distinguish between malignant and benign diseases for the diverse SUVmax, which is a crucial step in the diagnosis of mesothelioma [41]. Hyeon analysis demonstrated that PET had a 94.5% negative prediction value and 71% accuracy for excluding malignancy based on SUVmax. Malignancy resulted in a median 3.4 SUVmax compared to 1.7 in idiopathic cases in PET [42]. Our analysis shows median SUVmax at the level of 9.2 in the case of mesothelioma. Chang reported PET as a tool for prediction of response to steroid therapy in constrictive pericarditis [43]. To avoid nondiagnostic biopsy results, PET may help in the selection of optimal biopsy site [44]. According to the guidelines, fluorodeoxyglucose-PET/CT (FDG-PET/CT) should be considered for the diagnostic workup in patients with suspected myocarditis or pericarditis in whom echocardiography and cardiac magnetic resonance (CMR) are inconclusive for the clinical diagnosis [45]. Constriction features could be assessed in echocardiography or CMR leading to surgical biopsy; all the more the lack of such features should dispose clinicians to perform PET/CT in those inconclusive cases.

MRI is the best modality for the evaluation of the extension, the infiltration of the great vessels and cardiac wall, and the nature of the constrictive process; moreover, it can display pericardial involvement of mediastinal tumors, which is not apparent on CT scans [41]. Pericardial mesothelioma is homogeneously isointense on T1-weighted images, heterogeneous on T2-weighted images, and gadolinium-enhanced [46,47]. CMR has high accuracy in the exclusion of cardiac tumors; however, there have been reported cases with no abnormalities visible on MRI [24,48]. Some authors of analyzed articles reported CMR results with: irregularly thickened and strongly contrasted pericardium [49]; tissue characterization achieved with fat saturation T2-weighted imaging with hyperintense signal [21]; increased signal intensity on T1 with fat suppression, and increased signal intensity on T2 [50]; mass with low values on apparent diffusion coefficient (ADC) map of diffusion-weighted imaging (DWI) [51]; with late gadolinium enhancement and prolonged native T1 and T2 relaxation times, indicating increased vascularization [52]. Chetrit’s review recommended imaging-guided therapy of pericardial diseases assessing constrictive physiology in echocardiography and then evaluating T2W and late gadolinium enhancement (LGE) in CMR [53].

#### 4.3.3. Underutilization of Imaging in Clinical Practice

It occurs that, despite wide availability, the use of imaging methods is deficient. Our cohort revealed a pattern that inverts current guideline recommendations. Whereas biopsy was performed in 77.5% of patients, advanced non-invasive imaging was used considerably less frequently: CT in 77%, PET in 44.2%, and CMR in only 28.2%. According to ESC and contemporary expert recommendations, tissue biopsy should be reserved for cases in which multimodality imaging and cytological evaluation fail to establish a diagnosis [45]. The observed pattern therefore suggests that, in this cohort, invasive sampling preceded the full exhaustion of non-invasive diagnostic options—independent of any survival considerations.

The statistical associations between imaging modalities (CT, MRI, PET) and longer survival presented in Table 4 must be interpreted with great caution and do not support a causal benefit of imaging on outcome. These associations are highly susceptible to lead-time, selection, and immortal-time bias: patients with fulminant disease or acute hemodynamic collapse often die before advanced scans can be scheduled, while patients with more indolent courses survive long enough to complete an extended diagnostic workup. The same mechanism most likely explains the apparent association between misdiagnosis and longer survival, which reflects prolonged survival prior to definitive diagnosis rather than any true prognostic advantage.

Accordingly, we do not claim that PET/CT or CMR prolongs survival. Rather, on the basis of guideline-recommended diagnostic hierarchy—not on the basis of our survival data—advanced multimodality imaging may facilitate earlier recognition and diagnostic clarification in clinically suspicious cases, and could reduce reliance on invasive sampling as a first-line approach.

#### 4.3.4. Proposed Multimodality Imaging-Based Diagnostic Pathway

**Figure 5:**
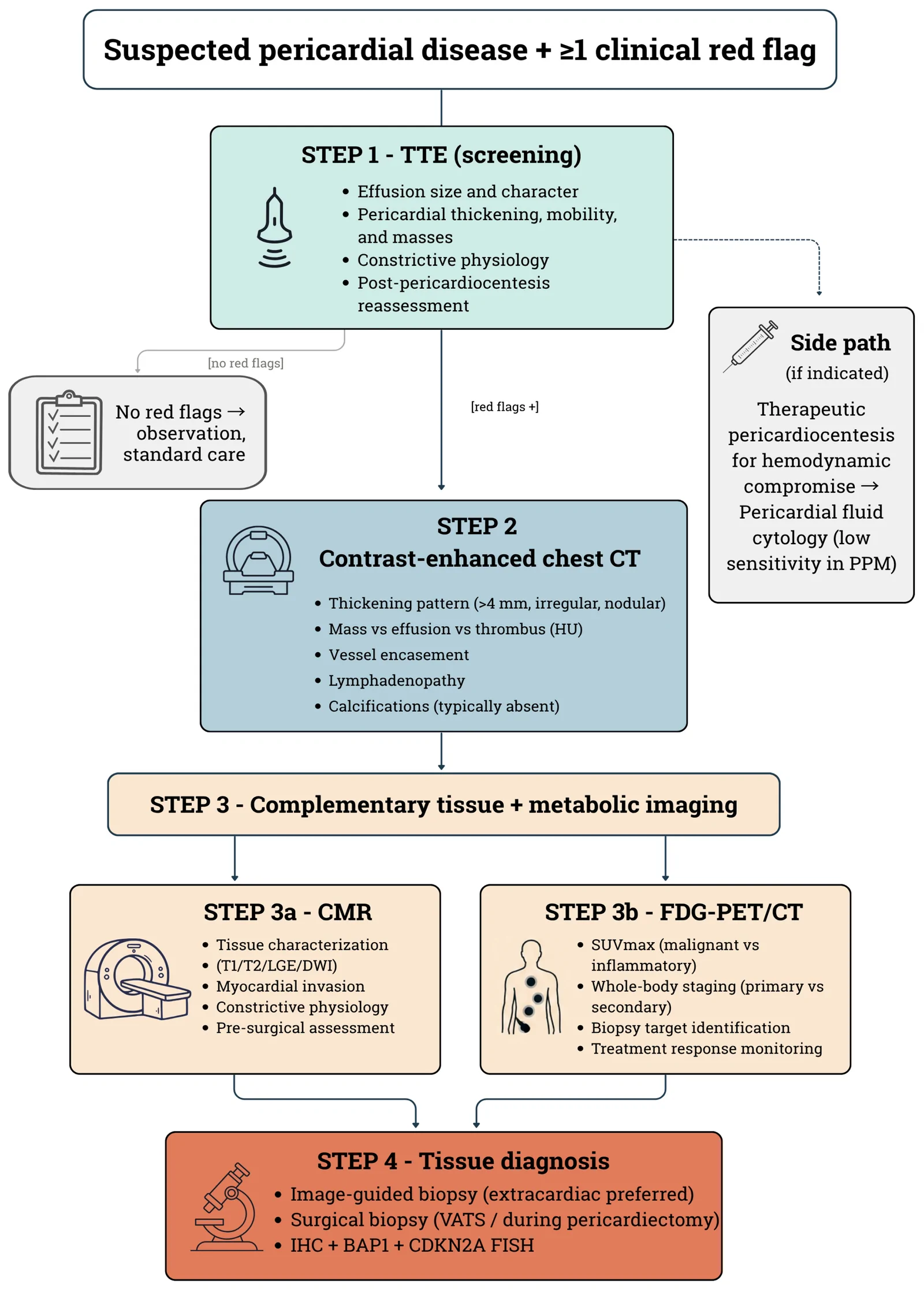
Proposed multimodality imaging-based diagnostic pathway for primary pericardial mesothelioma (PPM). Abbreviations: BAP1, BRCA1-associated protein 1; CDKN2A, cyclin-dependent kinase inhibitor 2A; CMR, cardiac magnetic resonance; CT, computed tomography; DWI, diffusion-weighted imaging; FDG-PET/CT, fluorodeoxyglucose positron emission tomography/computed tomography; FISH, fluorescence in situ hybridization; HU, Hounsfield units; IHC, immunohistochemistry; LGE, late gadolinium enhancement; PPM, primary pericardial mesothelioma; SUVmax, maximum standardized uptake value; T1, T1-weighted imaging; T2, T2-weighted imaging; TTE, transthoracic echocardiography; VATS, video-assisted thoracoscopic surgery.

Building on the evidence reviewed above [37,38,39,40,41,42,43,44,45,46,47,48,49,50,51,52,53], we propose a structured four-step diagnostic pathway in which each modality is assigned to the clinical question it answers best (Figure 5). Following TTE-based screening, contrast-enhanced chest CT forms the structural foundation of the diagnostic workup: irregular or nodular pericardial thickening exceeding 4 mm, Hounsfield unit-based discrimination of mass from effusion or thrombus, vessel encasement, mediastinal lymphadenopathy, and paradoxically the absence of pericardial calcifications collectively raise oncological suspicion and inform biopsy planning. CMR and FDG-PET/CT subsequently provide complementary rather than alternative information and remain markedly underutilized in current clinical practice (28.2% and 44.2% in our cohort, respectively). CMR enables superior tissue characterization, assessment of myocardial and epicardial invasion, evaluation of the pericardial–myocardial interface, and preoperative anatomical assessment. FDG-PET/CT contributes additional information beyond CMR in three clinically relevant areas: differentiation of malignant from inflammatory pericardial disease based on metabolic activity and SUVmax, whole-body staging to exclude secondary pericardial involvement, and identification of optimal biopsy targets. Importantly, extracardiac FDG-avid lesions may provide safer and diagnostically more accessible biopsy sites than direct pericardial sampling. The diagnostic pathway is completed by histopathological confirmation obtained through image-guided or surgical biopsy with immunohistochemical profiling supplemented by BAP1 assessment and CDKN2A fluorescence in situ hybridization where available. Therapeutic pericardiocentesis with fluid cytology functions as an adjunctive side path activated by hemodynamic compromise rather than as a standalone diagnostic step, reflecting the limited cytological yield observed in our cohort. The sequencing of modalities according to the clinical question is best suited to answer aims to maximize diagnostic yield while minimizing both diagnostic delay and the risk of non-diagnostic invasive procedures.

#### 4.3.5. Biopsy and Timing of Diagnosis

Pericardial biopsy may be considered in patients with suspected malignant pericarditis while imaging tools are exhausted [45]. The majority of specimens were taken during a pericardiectomy or a pericardial window procedure performed for therapeutic purposes. Only in 20 patients were there percutaneous or thoracoscopic biopsies performed as the only diagnostic approach, which occurred to be not optimal while those patients had significantly worse outcomes in Kaplan Maier survival analysis (*p* = 0.007). Some authors also struggled with biopsy results as non-diagnostic in both surgical and non-surgical approaches [54,55]. The diagnostic value of pericardial biopsy in metastatic neoplasms of the pericardium had an overall sensitivity of 57.69%, a specificity of 100%, a positive predictive value of 100%, and a negative predictive value of 87.06% [56]. Given these data, the question arises whether the diagnostic potential of imaging studies had actually been utilized before performing the biopsy, especially in view of the suboptimal use of both surgical and non-surgical biopsy techniques. Recurring or chronic pericarditis accompanied by persistent cardiovascular symptoms could justify early consideration of MRI or PET scanning. According to the guidelines, surgical pericardiectomy is recommended in patients with chronic pericardial constriction or persistent constrictive pericarditis despite medical therapy, to improve symptoms and survival. The observed association between constrictive physiology and poor survival highlights the potential benefit of establishing a diagnosis prior to advanced fibrotic remodeling. In our population, 31.7% of patients developed constrictive pericarditis, which correlated with significantly shorter survival on Kaplan-Meier analysis. Advanced constriction complicates pericardiectomy due to dense calcifications and the risk of ventricular free wall injury, and delayed surgery beyond 6 months increases mortality [57]. Diagnosing PPM during acute or recurrent pericarditis episodes targets a phase prior to the development of chronic constrictive changes.

#### 4.3.6. A Unified Disease-Evolution Model for PPM

**Figure 6:**
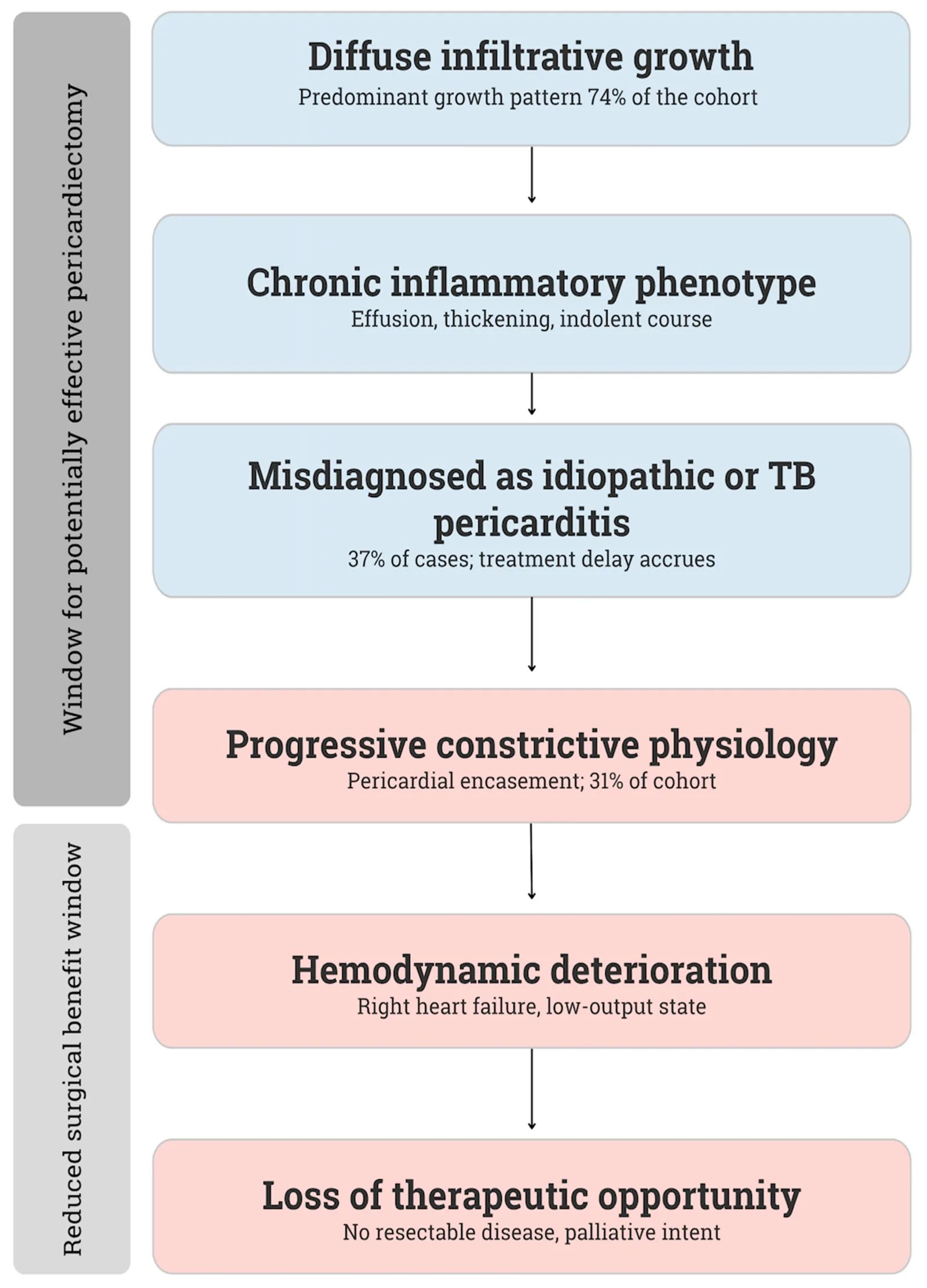
Proposed disease-evolution model for PPM. Similarly, solid or tumor-like pericardial masses were more often regarded as potentially neoplastic than uniform pericardial thickening, directing further diagnostic evaluation toward malignancy. Both phenotypes show that the prognostic penalty of diffuse disease is mediated chiefly by diagnostic delay rather than by tumor biology alone: whenever the chronic inflammatory pathway is bypassed, outcomes improve.

Considered together, the associations reported above describe a coherent disease-evolution sequence rather than a list of independent risk factors. Diffuse infiltrative growth—the dominant anatomic pattern in our cohort—produces a chronic inflammatory phenotype consisting of recurrent or persistent effusion, uniform pericardial thickening, and an indolent course that is clinically indistinguishable from idiopathic, viral, or tuberculous pericarditis. In our analysis, both diffuse neoplastic process and constrictive pericarditis were associated with significantly shorter survival on Kaplan-Meier curves [Figure 2 and Figure 3]; it also seems to be linked in causal connection. In the analyzed population, 31.7% of patients developed constrictive pericarditis, and Kaplan-Meier analysis demonstrated significantly better survival among those without constrictive pericarditis. Notably, these subgroups also demonstrated a prolonged diagnostic process, which may partially explain the unfavorable outcomes. The slow progression and nonspecific clinical presentation of these forms often delayed the establishment of a definitive diagnosis. As a result, patients frequently reached a state of hemodynamic decompensation before causal treatment was initiated, reducing the potential therapeutic benefit. We therefore propose that constrictive pericarditis is the pivotal clinical node of this disease, linking anatomy (diffuse infiltration) to misdiagnosis (mimicry of benign disease) and ultimately to outcome (hemodynamic decompensation before causal treatment) **(**Figure 6**)**.

Misdiagnosis as a predictable consequence of mimicry. In this framework, misdiagnosis is not a clinician error but a predictable consequence of the tumor’s anatomic behavior. Older age being associated with longer survival was probably related to misdiagnosis, which occurred significantly more often among younger patients—with a median age of 45 years in those with misdiagnosis, compared to 58.5 years in patients without it. Fernandes et al. reported a 30-year-old woman initially suspected of having decompensated heart failure secondary to Hepatitis C virus (HCV) infection, while Dung et al. described a case initially diagnosed as Demons-Meigs syndrome [58,59]. Pericardial mesothelioma was often misdiagnosed as tuberculous pericarditis. Yan et al. reported a case of a patient treated for 12 months for presumed tuberculous pericarditis, and Zhang et al. reported a case of a patient in whom the correct diagnosis was made only after pericardiectomy for presumed tuberculous constrictive pericarditis [14,60]. Similarly, in cases where the disease was initially misdiagnosed as a systemic condition, malignancy was suspected only after the development of a constrictive process. For example, Mensi et al. reported a 45-year-old woman initially misdiagnosed with systemic lupus erythematosus, with the correct diagnosis established after pericardiectomy for constrictive pericarditis [61]. The observed association of misdiagnosis with longer survival from symptom onset likely reflects a diagnostic timing bias rather than a true prognostic effect, as both factors may indicate a prolonged interval between initial symptoms and definitive diagnosis, artificially extending the measured survival time.

The constrictive node and the closing therapeutic window are described below. The constrictive node also defines the therapeutic window. In advanced constrictive pericarditis, pericardiectomy becomes more difficult to perform due to multiple calcifications and the risk of ventricular free wall injury. This is supported by the study by Vistarini, in which patients operated within 6 months after the onset of symptoms showed a lower risk of mortality [57]. According to the guidelines, surgical pericardiectomy is recommended in patients with chronic pericardial constriction or persistent constrictive pericarditis despite medical therapy, to improve symptoms and survival. As the constrictive process worsens a patient’s prognosis in mesothelioma, it would be reasonable to perform resection of the neoplasm before constriction develops. Such a perspective encourages clinicians to consider the diagnosis of pericardial mesothelioma at the stage of acute or recurrent pericarditis, before chronic and constrictive process starts. Recurring or chronic pericarditis and a history of persistently present cardiovascular symptoms should prompt clinicians to perform MRI or PET scanning. It would require FDG-PET/CT assessment after exclusion of diagnosis and settle red flags suggesting a neoplastic process. These features are poor prognostic predictors and include: high fever (>38 °C; hazard ratio [HR], 3.6), unusual for uncomplicated viral or idiopathic pericarditis, subacute course (without acute onset or without pericarditis chest pain; HR, 4), large pericardial effusion (>20 mm as the largest telediastolic echo-free space) or cardiac tamponade at presentation (HR, 2.1–2.5), and failure to respond to empiric anti-inflammatory therapy with aspirin or nonsteroidal anti-inflammatory drugs (NSAIDs; HR, 2.5–5.5). Female sex is associated with an increased risk of developing complications or pericarditis of nonidiopathic etiology (HR, 1.6–1.7) [62]. Those risk factors correspond with risk factors of constrictive pericarditis, which is the most severe state during chronic pericarditis. Patients who developed constrictive pericarditis during follow-up showed a higher frequency of specific features compared with those without such evolution: fever >38 °C incessant course, nonidiopathic/nonviral etiology, large pericardial effusion, cardiac tamponade, and aspirin/nonsteroidal anti-inflammatory drug failure at 1 week [63]. These markers of complicated or nonidiopathic pericarditis overlap with the very features that predict subsequent constrictive evolution, and in this context should be read as red flags for occult malignancy.

An argument supporting this model is presented below. One observation in our data initially appeared to contradict the model but in fact reinforces it. Interestingly, longer survival was associated with the presence and amount of pericardial effusion [Table 8]. Patients with large pericardial effusions were diagnosed earlier and had longer survival. Such patients were highly symptomatic and raised suspicion of specific types of pericarditis. Large effusions enabled pericardiocentesis and cytological analysis of the obtained fluid, which established the diagnosis in several patients; for example, Wu et al. reported a patient initially treated for tuberculous pericarditis with massive pericardial effusion, in whom cytological examination revealed the presence of mesothelial cells [64]. Peralta-Amaro et al. described a patient with severe pericardial effusion in whom a detailed diagnostic process led to an early biopsy-based diagnosis and a survival of 38 months [5]. The importance of performing cytological examination whenever pericardial fluid is obtained was underscored in the report by Kayotta, which described patients who had not undergone cytology initially; however, retrospective analysis after histological confirmation based on biopsy revealed mesothelioma [65].

### 4.4. Therapeutic Approach

There is no standardized treatment protocol for primary pericardial mesothelioma due to its rarity and the lack of prospective trials [18]. Management is guided by case reports, small series, and by analogy to pleural mesothelioma. Multimodality treatment, combining surgery, chemotherapy, and radiotherapy, is considered the potential standard for resectable pleural mesothelioma and currently offers the best chance for prolonged survival [7]. Surgical treatment of pericardial mesothelioma is highly challenging. Complete resection is rarely feasible because the tumor typically infiltrates the myocardium and invades adjacent structures. In rare cases where the disease presents as a localized mass and full resection is possible, long-term survival has been reported [41]. Unfortunately, in the majority of patients, the diagnosis is made only at an advanced stage, which significantly limits the possibility of performing radical resection with an R0 margin [66]. Most often, surgery has a palliative role. Surgical approaches, including pericardiectomy, are chiefly employed in pericardial mesothelioma to alleviate hemodynamic compromise from effusion or constriction and achieve partial tumor reduction. In addition, creating a pericardial window may facilitate symptom control and establish a controlled access point for administering and monitoring intrapericardial therapies, serving as a strategic bridge toward multimodal treatment approaches [32,41]. In some case reports, surgery has not demonstrated a statistically significant survival benefit in PPM, while systemic chemotherapy—most commonly platinum-pemetrexed combinations or regimens including doxorubicin, vincristine, and cyclophosphamide—has shown some efficacy [22]. A historical review of 103 PPM cases demonstrated a survival benefit exclusively in patients receiving chemotherapy, particularly platinum-based regimens with or without pemetrexed (median survival 13 vs. 0.5 months [8]. Combination chemotherapy with cisplatin and pemetrexed has been shown to prolong survival in pleural mesothelioma and is widely regarded as the standard first-line treatment for primary pericardial mesothelioma [30,67]. In certain cases, carboplatin is substituted for cisplatin in combination with pemetrexed to reduce cardiac toxicity and overall treatment burden [30]. Such retrospective observations must be interpreted with caution due to prominent selection and treatment eligibility biases. Randomized studies in pleural mesothelioma demonstrate that the addition of antifolate agents to platinum-based regimens, and the use of bevacizumab in patients unsuitable for curative surgery, increase objective response rates and prolong progression-free and overall survival [68]. In one reported case, the addition of bevacizumab to the cisplatin-pemetrexed regimen produced metabolic disease stabilization on PET-CT, suggesting a durable response. Therefore, bevacizumab warrants consideration as a component of second-line or adjunctive regimens in select patients [69]. Across multiple statistical tests and analyses, chemotherapy consistently emerged as a factor associated with longer survival (Table 6 and Table 7). Nevertheless, interpreting this finding as a straightforward survival improvement is overly simplistic. These retrospective data are heavily constrained by selection, survivor and treatment eligibility biases, as patients receiving therapy inherently survived long enough to undergo a complex diagnostic pathway, possessed a confirmed diagnosis, preserved performance status, and less hemodynamic compromise. Additionally, case report datasets suffer from publication bias, disproportionately reporting successful outcomes over rapid fatalities. Because no randomized trials have been conducted specifically in primary pericardial mesothelioma, clinicians frequently extrapolate these pleural-based strategies to the pericardial setting [68]. In the chemotherapy-treated cohort, poorer performance status correlated with markedly reduced survival, underscoring that preserved functional status (PS 0–1) identifies patients most likely to derive meaningful benefit from systemic therapy (median OS-34 vs. 4 months for PS 0 and PS 4, respectively) [70]. Consequently, chemotherapy and surgery must be viewed within a real-world framework: surgery functions primarily as a diagnostic or hemodynamic stabilization tool (e.g., drainage, pericardiectomy) to prevent acute decompensation, whereas systemic therapy is often unfeasible or delayed in patients presenting with advanced, diffuse infiltrative disease.

Radiotherapy has a limited role in PPM and is usually used only as adjuvant therapy after incomplete resection [30]. Current evidence is predominantly derived from descriptive case reports and series, and the absence of prospective trials precludes definitive evaluation of this intervention’s therapeutic benefits [22,69]. Accordingly, radiation therapy should be regarded primarily as an adjunctive treatment modality aimed at symptom palliation, preservation of physical function, and mitigation of potential complications [71]. In our cohort, three patients received radiotherapy as part of a trimodal approach (surgery, adjuvant chemotherapy, radiotherapy), resulting in a substantially longer median overall survival (70.3 months) compared with patients treated without trimodality therapy (8.2 months) [18]. While multimodal therapy is typically extrapolated from pleural disease standards [72], our data suggest that combining surgery, systemic therapy, and radiotherapy also yields better outcomes in pericardial mesothelioma. Patient selection for this aggressive management requires multidisciplinary team evaluation [73]. Since specific cases demonstrate complete symptomatic remission and an uneventful course [7], a multimodal approach should be considered when resection is feasible. To better reflect the modern therapeutic landscape, systemic treatments in our cohort were further stratified into immune checkpoint inhibitor regimens and targeted combinations (Table 5). Systemic chemotherapy and immunotherapy choices increasingly rely on immune checkpoint inhibitors (ICIs) [74]. Large-scale trials, such as CheckMate 743, established ICIs for unresectable pleural disease but excluded patients with primary pericardial involvement, limiting direct extrapolation [28,75]. However, individual PPM cases in our dataset offer direct clinical observations. In one case, nivolumab monotherapy achieved a significant clinical and radiological response despite the patient’s renal dysfunction and poor performance status [49]. In another, switching to combination nivolumab and ipilimumab led to a marked response correlating with moderate (30%) PD-L1 expression, highlighting the potential role of biomarker testing in PPM [76]. Cases utilizing ICIs in subsequent lines further demonstrate that long-term disease control is achievable [28,77]. Because current evidence rests primarily on case reports, management must be tailored to the individual. Experimental strategies—including intrapericardial chemotherapy, stereotactic irradiation, and targeted growth-factor pathway inhibitors—remain exploratory [31]. For the majority of PPM patients presenting with advanced disease, therapeutic choices are governed by baseline performance status, comorbidities, and the clinical goal of balancing treatment toxicity against quality of life [78]. To better reflect the modern therapeutic landscape, systemic treatments were further stratified into immune checkpoint inhibitor regimens and targeted combinations (Table 5).

### 4.5. Study Limitations

This study has several important limitations related to the nature of the available data. First, the analysis was based exclusively on published case reports, which represent a low level of evidence and are inherently subject to multiple sources of bias. In particular, selective reporting (reporting bias) is likely, as authors may preferentially describe unusual, severe, or clinically interesting features while omitting less remarkable findings. As a result, the dataset may not fully reflect the true clinical spectrum of primary pericardial mesothelioma. Many authors reported follow-up periods shorter than 12 months and stated that patients were alive at the end of the observation period. This may have led to an overestimation of survival outcomes. Furthermore, the heterogeneity and incompleteness of available data limited the possibility of performing standardized comparative analyses. This study provides the largest pooled analysis of individual patient data in PPM to date, although the results are limited by the inherent biases of case report-based evidence. Beyond these methodological constraints, how primary mesothelioma arises in the pericardium remains a fundamental, unresolved conceptual issue in PPM biology. To achieve a more accurate understanding of clinical characteristics, diagnostic pathways, treatment outcomes, prognosis and underlying biological mechanisms in patients with pericardial mesothelioma, a prospective multicenter study with comprehensive and standardized data collection is warranted. The findings should therefore be interpreted with caution and considered primarily exploratory, highlighting potential clinical patterns rather than establishing definitive causal relationships.

## 5. Conclusions

The prognosis for primary pericardial mesothelioma is unfavorable in practice, as repeatedly confirmed by contemporary publications. One-year survival is low (22%), reflecting the rapid and aggressive course of the disease [33]. Median survival after diagnosis is approximately six months, with individual series reporting a range of three to ten months [1,79]. Even with chemotherapy, radiotherapy, or surgery, median survival rarely exceeds six to ten months, underscoring the uniformly poor prognosis associated with this site [80]. The tumor is most often detected at an advanced stage, limiting the options for definitive treatment [79]. The main direct causes of death are cardiac tamponade and heart failure resulting from localized pericardial involvement [32]. Retrospective data associate timely identification and individualized management with prolonged survival in select cases [8]. These findings remain exploratory due to retrospective design limitations. Although advanced imaging and laboratory assays aid differential diagnosis [81], low disease incidence precludes randomized controlled trials. Prospective multicenter registries are necessary to validate these observations and guide management. Finally, future research must also focus on how primary mesothelioma develops in the pericardium, as its exact pathogenesis remains insufficiently understood.

## Figures and Tables

**Table 1:** Baseline characteristics.

Characteristic	Number (%) or Median (Range)
Age	53.6 (17–85) IQR (42–66)
Age not reported	*n* = 3
Sex (*n* = 120 cases)	
Men	55.8% (*n* = 67)
Women	44.2% (*n* = 53)
Exposures (*n* = 120 cases)	
Asbestos exposure * 71 authors mentioned if patient had exposure or not	11.67% (*n* = 14)
Comorbidities (*n* = 120 cases)	
Cardiovascular comorbidities	20% (*n* = 24)
Other comorbidities	21.7% (*n* = 26)
Past tumors	13.3% (*n* = 16)

**Table 2:** Clinical presentation, histologic subtype, and tumor characteristics.

Clinical Manifestation (*n* = 120 Cases)	
Constrictive pericarditis	31.7% (*n* = 38)
Any sign of constrictive physiology	40% (*n* = 48)
Pericardial effusion	75.8% (*n* = 91)
Bloody effusion	31.7% (*n* = 38)
Median amount of fluid in imaging	19 mm IQR (13,8–20)
Diffuse pericardial thickening	74.2% (*n* = 89)
Recurrent/refractory pericarditis	33.3% (*n* = 40)
Histologic Subtype (*n* = 88 cases)	
Not determined	26.6% (*n* = 32)
Epithelioid	67.05% (*n* = 59)
Sarcomatoid	11.36% (*n* = 10)
Biphasic	17.05% (*n* = 15)
Others	4.55% (*n* = 4)
Type of neoplastic process (*n* = 120)	
Localized tumor	30.8% (*n* = 37)
Diffuse process	74.2% (*n* = 89)
Both types	5% (*n* = 6)
Median tumor volume in imaging	213 cm^3^ IQR (102–480)
Median pericardium thickening	19 mm IQR (13.8–20)
Metastases (*n* = 22 cases)	
Local metastases	10.8% (*n* = 13)
Distal metastases	13.5% (*n* = 17)
Both	5% (*n* = 6)

**Table 3:** Factors associated with longer diagnostic processes.

Factors Associated with Longer Diagnostic Time (from First Symptoms to Diagnosis)			
Factor	Median diagnostic time (+)	Median diagnostic time (−)	*p*-value
Diffuse neoplastic process	5.5	2	0.045
Constrictive pericarditis	8	3	0.003
Misdiagnosis	6.5	3	0.0012
Positive pericardial fluid cytology result	2.65	6	0.02
Thoracic biopsy in diagnostic process	6	3	0.015

**Table 4:** Utility of diagnostic methods, direct method of diagnosis and incidence of misdiagnosis.

Diagnostic Workup (*n* = 120)	
Cytology test of pericardial fluid	48.3% (*n* = 58)
Positive result of cytology test	37.9% (*n* = 22)
Any type of biopsy	77.5% (*n* = 93)
Surgical biopsy	63.3% (*n* = 76)
Percutaneous biopsy	6.6% (*n* = 8)
Endoscopic biopsy (VATS, endobronchial)	7.5% (*n* = 9)
CT	77% (*n* = 93)
MRI	28.2% (*n* = 34)
PET	44.2% (*n* = 53)
Median SUVmax in PET (*n* = 17)	9.2 IQR (6.7–17.8)
Method that settled diagnosis (*n* = 120)	
Non-surgical biopsy	20% (*n* = 24)
Surgical biopsy/during pericardiectomy	68.3% (*n* = 82)
Autopsy	6.7% (*n* = 8)
Pericardial fluid cytology test	3.3% (*n* = 4)
No information	1.7 (*n* = 2)
Misdiagnosis	
Misdiagnosis	34.2% (*n* = 41)
Treatment for idiopathic pericarditis	16.7% (*n* = 20)
Treatment adequate for misdiagnosis	16.7% (*n* = 20)

**Table 5:** Treatment.

Treatment (*n* = 120)				
Radiation			8.3% (*n* = 10)	
Chemotherapy			38.3% (*n* = 46)	
Surgery			65% (*n* = 78)	
Total number of surgical procedures (*n* = 85)				
>1 procedures			5.8% (*n* = 7)	
Curative/localized resection			9.4% (*n* = 8)	
Debulking surgery			34.1% (*n* = 29)	
Pericardiectomy for constrictive physiology			32.9% (*n* = 28)	
Window/Relief procedures			23.5% (*n* = 20)	
Type of chemotherapy (*n* = 46)				
Single-modality therapy			8.7% (*n* = 4)	
Bimodality therapy			60.9% (*n* = 28)	
Trimodality therapy			30.4% (*n* = 14)	
Type of pharmacotherapy (*n* = 47)	*n*	% of treated patients	Representative regimens	Reported outcomes
1. Nivolumab + ipilimumab	2	2.7%	Nivolumab + Ipilimumab (1st line/adjuvant)	Median survival ~6 months; initial stabilization followed by systemic progression.
2. Pembrolizumab-based therapy	0	0.0%	N/A	N/A
3. Chemo- immunotherapy	1	1.4%	Carboplatin + Pemetrexed + Pembrolizumab	Tumor regression, complete relief of symptoms, alive and on maintenance ICI.
4. Platinum + pemetrexed + bevacizumab	2	2.7%	Cisplatin + Pemetrexed + Bevacizumab	Median survival ~10.5 months; stable disease and symptom relief achieved.
5. Salvage immunotherapy	3	4.1%	Pembrolizumab (post-chemo); Nivolumab + Ipilimumab (post-chemo)	Median survival ~36 months; highly variable responses ranging from rapid progression to >6 years of disease control.

**Table 6:** Survival.

Survival and Diagnostic Time	
Median diagnostic time (from first symptoms to diagnosis)	4 months IQR (1–11)
Median survival time for first symptoms	9 months IQR (2.5–17)
Median survival time from diagnosis	7.4 months IQR (1.75–13.5)

**Table 7:** Univariable analysis of factors influencing survival from the onset of symptoms.

Factors Associated with Shorter Survival from First Symptoms			
Factor	Median survival time (−)	Median survival time (+)	*p*-value
Cardiovascular comorbidities	3	12	0.008
Past tumors	0.75	9	0.036
Factors associated with longer survival from first symptoms			
Initial treatment for idiopathic or different etiology pericarditis	14.25	5	0.008
PET use in diagnostic process	14	6	0.04
CT use in diagnostic process	9.125	1,5	0.02
Chemotherapy	16	4,75	<0.001

**Table 8:** Factors associated with longer survival from diagnosis.

Factors Associated with Longer Survival from Diagnosis			
Factor	Median survival time (−)	Median survival time (+)	*p*-value
Sex [♂ = (−); ♀ = (+)]	4	9	0.04
Effusion	5.5	8	0.04
Positive cytology result	6	12	0.03
Surgery	3	8	0.06
Chemotherapy	3	10	<0.001

**Table 9:** Multivariable Cox adjusted model.

Variable	aHR (95% CI)	*p*-Value
Age ≥ 60	0.82 (0.43–1.57)	0.557
sex M = 1 K = 0	1.63 (0.88–3.05)	0.122
Non-epithelioid subtype	0.93 (0.47–1.81)	0.824
Diffused disease	2.08 (1.02–4.25)	0.045
Surgery	0.88 (0.44–1.75)	0.713
Chemotherapy	0.40 (0.21–0.77)	0.006

## Data Availability

The data supporting the findings of this study are openly available in Zenodo at https://doi.org/10.5281/zenodo.19498485. Accessed: 29 May 2026.

## Supplementary Materials

The following supporting information can be downloaded at: https://www.mdpi.com/article/10.3390/cancers18162583/s1, File S1: PRISMA 2020 Checklist; File S2: PRISMA 2020 for Abstracts Checklist; Table S3: Bibliographic characteristics of included studies; Table S4: Risk of Bias assessment; Table S5: Kaplan-Meier survival analysis from symptom onset; Table S6: Kaplan-Meier survival analysis from diagnosis; Table S7: Logistic regression analysis of survival from symptom onset; Table S8: Logistic regression analysis of survival from diagnosis; Table S9: Univariable Cox proportional hazards regression; Table S10: Performance and diagnostic metrics of the multivariable Cox proportional hazards model; Table S11: Assessment of the proportional hazards assumption using Schoenfeld residuals; Figure S1: Kaplan-Meier survival curve according to surgical treatment; Figure S2: Kaplan-Meier survival curve according to chemotherapy.
