# Supplementary material for: Prognostic Factors and Survival in Pericardial Mesothelioma: A Systematic Review and Quantitative Analysis
Source: Cancers (Basel). 2026 Aug 11;18(16):2583. doi: 10.3390/cancers18162583 (PMC13511537; doi:10.3390/cancers18162583)
Supplement: Supplementary file 1 [file cancers-18-02583-s001.zip › Supplementary material - Kaplan-Maier S5 and S6.pdf]

Supplementary Table S5. Kaplan-Meier survival analysis and log-rank test (from symptom onset).

Variable	N	Group sizes	χ^2 (df=1)	p-value
Sex	41	25 vs 16	1.94	0.053
Azbest exposure	37	14 vs 23	1.26	0.207
Cardiovascular comorbidities	43	32 vs 11	2.54	**0.011**
Past tumors	43	5 vs 38	1.69	0.090
Other comorbidities	43	14 vs 29	1.01	0.311
Localised mesothelioma	43	29 vs 14	0.48	0.629
Diffused mesothelioma	43	33 vs 10	0.74	0.457
Constrictive pericarditis	43	12 vs 31	1.63	0.103
Presence of effusion	43	10 vs 33	1.76	0.079
Cytology test	43	26 vs 17	0.94	0.350
Cytology result	43	14 vs 29	0.39	0.695
Misdiagnosis	43	27 vs 16	1.93	0.054

Treated as idiopathic pericarditis	43	35 vs 8	0.78	0.435
Treated as specific type pericarditis	43	33 vs 10	2.94	**0.003**
Thoracic biopsy	43	22 vs 21	1.01	0.315
PET	43	20 vs 23	1.68	0.092
MRI	43	28 vs 15	0.34	0.735
CT	43	7 vs 36	1.46	0.145
Local metastasis	43	36 vs 7	1.11	0.267
Distant metastases	43	37 vs 6	0.98	0.328
Surgery	43	18 vs 25	0.15	0.878
Chemotherapy	43	26 vs 17	3.56	**0.00038**
Immunotherapy	*43*	*40 vs 3*	*2.12*	***0.034***
Radiotherapy	*43*	*40 vs 3*	*2.35*	***0.019***

Supplementary Table S6. Kaplan-Meier survival analysis and log-rank test (from diagnosis).

**Variable**	**N**	**Group sizes**	**χ^2 (df=1)**	**p-value**
Sex	71	38 vs 33	1.29	0.198
Azbest exposure	71	62 vs 9	0.27	0.786
Cardiovascular comorbidities	71	57 vs 14	0.49	0.624
Past tumors	71	5 vs 66	1.38	0.168
Other comorbidities	71	18 vs 53	1.96	**0.0496**
Localised mesothelioma	71	45 vs 26	1.82	0.069
Diffused mesothelioma	71	49 vs 22	2.12	**0.034**
Constrictive pericarditis	71	20 vs 51	2.51	**0.012**
Presence of effusion	71	20 vs 51	1.13	0.259
Cytology test	71	48 vs 23	0.61	0.544
Cytology result	71	23 vs 48	0.19	0.851
Misdiagnosis	71	44 vs 27	1.33	0.185
Treated as chronic idiopathic pericarditis	71	57 vs 14	1.84	0.065

Treatment as for other specific pericarditis type	71	10 vs 61	1.62	0.105
Diagnosis confirmed by surgical biopsy	71	52 vs 19	1.53	0.127
Diagnosis confirmed by percutaneous biopsy	71	56 vs 15	2.58	**0.010**
Thoracic biopsy	71	35 vs 36	1.67	0.095
PET	71	30 vs 41	1.21	0.227
MRI	71	51 vs 20	0.81	0.416
CT	71	11 vs 60	0.98	0.328
Local metastasis	71	64 vs 7	1.65	0.098
Distant metastasis	71	61 vs 10	0.87	0.387
Surgery	71	42 vs 29	1.37	0.171
Chemotherapy	71	37 vs 34	3.46	**0.00053**
Immunotherapy	71	67 vs 4	0.26	0.794
Radiotherapy	71	64 vs 7	0.67	0.501
