# Supplementary material for: Prognostic Factors and Survival in Pericardial Mesothelioma: A Systematic Review and Quantitative Analysis
Source: Cancers (Basel). 2026 Aug 11;18(16):2583. doi: 10.3390/cancers18162583 (PMC13511537; doi:10.3390/cancers18162583)
Supplement: Supplementary file 1 [file cancers-18-02583-s001.zip › Supplementary material Cox regression S9-S11.pdf]

Supplementary table S9

Univariable Cox proportional hazards regression for overall survival.

Variable	N	Events	Exposed	HR (95% CI)	p-value
Age ≥ 60 years	68	47	26	1.27 (0.70-2.27)	0.431
Male sex	70	49	37	1.40 (0.79-2.46)	0.245
Non-epithelioid subtype	70	49	42	1.22 (0.68-2.19)	0.504
Diffuse pericardial involvement	70	49	48	1.94 (0.99-3.82)	0.055
Surgery	70	49	49	0.70 (0.38-1.28)	0.244
Chemotherapy	70	49	34	0.37 (0.20-0.66)	<0.001

HR = hazard ratio; CI = confidence interval. N = number of patients analyzed for each predictor; Events = number of deaths; Exposed = number of patients with the predictor coded as 1.

Supplementary Analysis of the Multivariable Cox Proportional Hazards Model

The performance and assumptions of the multivariable Cox proportional hazards model were evaluated using several complementary metrics. Model discrimination was assessed using Harrell's concordance index (C-index), while overall model significance was evaluated using likelihood ratio, Wald, and score tests. Model complexity was summarized using the Akaike Information Criterion (AIC). The proportional hazards assumption was assessed using Schoenfeld residuals for individual covariates and globally.

The final model included 68 patients and 47 events, corresponding to 7.83 events per variable. The model demonstrated moderate discriminative ability with a Harrell's C-index of 0.699. Overall model performance was statistically significant according to the likelihood ratio test (p = 0.0097), Wald test (p = 0.0124), and score test (p = 0.0079).

Assessment of proportional hazards assumptions revealed evidence of non-proportional hazards for sex (p = 0.027), surgery (p = 0.012), and chemotherapy (p = 0.029). The

global Schoenfeld residual test was also significant (p = 0.021), indicating that the proportional hazards assumption was not fully satisfied. Consequently, hazard ratios for these variables should be interpreted as average effects over the follow-up period rather than strictly constant effects over time.

Supplementary Table S10
Performance and diagnostic metrics of the multivariable Cox proportional hazards model

Metric	Value
Patients included	68
Events	47
Events per variable (EPV)	7.83
Harrell's C-index	0.699
Standard error of C-index	0.041
Likelihood ratio χ^2	16.88
Likelihood ratio p-value	0.0097
Wald χ^2	16.27
Wald p-value	0.0124
Score (log-rank) χ^2	17.39
Score (log-rank) p-value	0.0079
Akaike Information Criterion (AIC)	327.5
Global Schoenfeld test p-value	0.021

Supplementary Table S11
Assessment of the proportional hazards assumption using Schoenfeld residuals

Variable	Chi_square	P_value
Age ≥60 years	2.07	0.150
Male sex	4.88	0.027
Non-epithelioid subtype	0.53	0.467
Diffuse disease	1.17	0.279

Surgery	6.27	0.012
Chemotherapy	4.78	0.029
Global test	14.94	0.021
