# Supplementary material for: Prognostic Factors and Survival in Pericardial Mesothelioma: A Systematic Review and Quantitative Analysis
Source: Cancers (Basel). 2026 Aug 11;18(16):2583. doi: 10.3390/cancers18162583 (PMC13511537; doi:10.3390/cancers18162583)
Supplement: Supplementary file 1 [file cancers-18-02583-s001.zip › Supplementary Table S3.pdf]

Supplementary Table S3.

Article number	First Author	Reference
1	Edel	Edel JP, Balink H Constrictive pericarditis, malignant mesothelioma, 18F-FDG PET/CT Clin Nucl Med 2019 44(1) 55-5610.1097/RLU.0000000000002349
2	Li	Li X, Lu R, Zhao Y, Wang F, Shao G 18F-fluorodeoxyglucose imaging of primary malignant pericardial mesothelioma with concurrent pericardial and pleural effusions and bone metastasis: a case report Mol Clin Oncol 2018 8(6) 725-72810.3892/mco.2018.1604
3	Jodati	Jodati A, Kazemi B, Safaei N, Toufan M A ball in the heart: an interesting discovery in a very rare cardiac tumor J Cardiovasc Thorac Res 2013 5(2) 77-80 10.5681/jcvtr.2013.017
4	Lee	Lee MJ, Kim DH, Kwan J, Park KS, Shin SH, Woo SI, et al A case of malignant pericardial mesothelioma with constrictive pericarditis physiology misdiagnosed as pericardial metastatic cancer Korean Circ J 2011 41(6) 338-34110.4070/kcj.2011.41.6.338
5	Aga	Aga F, Yamamoto Y, Norikane T, Nishiyama Y A case of primary pericardial mesothelioma detected by 18F-FDG PET/CT Clin Nucl Med 2012 37(5) 522-523 10.1097/RLU.0b013e3182478c13
6	Fukasawa	Fukasawa N, Agemi Y, Shiba A, Aga M, Hamakawa Y, Miyazaki K, et al A case of slowly progressive malignant pericardial mesothelioma suggesting the involvement of BAP1 loss Respirol Case Rep 2022 10(7) e0100410.1002/rcr2.1004
7	Feng	Feng X, Zhao L, Han G, Cheng G, Xu J A case report of an extremely rare and aggressive tumor: primary malignant pericardial mesothelioma Rare Tumors 2012 4(2) e21 10.4081/rt.2012.e21
8	Banišauskaitė	Banišauskaitė A, Jankauskas A, Šarauskas V, Aržanauskaitė M A case report of malignant primary pericardial mesothelioma with atypical imaging appearance: multimodality imaging with histopathological

		correlation Eur Heart J Case Rep 2020 4(2) 1-5 10.1093/ehjcr/ytaa034
9	Kim	Kim JS, Lim SY, Hwang J, Kang EJ, Choi YJ A case report of primary pericardial malignant mesothelioma treated with pemetrexed and cisplatin J Korean Med Sci 2017 32(11) 1879-1884 10.3346/jkms.2017.32.11.1879
10	Wang	Wang X, Ren W, Xiao Y, Li J, Zhou K A giant, well-differentiated papillary mesothelioma of the left atrioventricular groove: case report and brief review of the literature J Clin Ultrasound 2019 47(9) 564-567 10.1002/jcu.22730
11	Betancor	Betancor J, Rodriguez ER, Smedira NG, Kalahasti V, Phelan D, Lytle BW, et al Constrictive pericarditis due to primary pericardial mesothelioma CASE (Phila) 2017 1(1) 17-22 10.1016/j.case.2016.11.005
12	Van de Bruaene	Van de Bruaene C, Crapé L, Surmont V Constrictive pericarditis caused by primary pericardial mesothelioma Heart 2017103(13) 1053 10.1136/heartjnl-2016-311101
13	Ahmad	Ahmad SDS Primary malignant pericardial mesothelioma: a case report J Surg Case Rep 2024 2024(5) rjae27910.1093/jscr/rjae279
14	Cui	Cui F, Hu Y, Li Y A rare case of primary malignant pericardial mesothelioma diagnosed with pericardiotomy Heart Surg Forum 2022 25(6) E840-E842 10.1532/hsf.5047
15	Fernandes	Fernandes R, Nosib S, Thomson D, Baniak N A rare cause of heart failure with preserved ejection fraction: primary pericardial mesothelioma masquerading as pericardial constriction BMJ Case Rep 2014 2014 bcr201320319410.1136/bcr-2013-203194
16	Bokma	Bokma JP, Rijlaarsdam MA, Ambarus CA, Bindraban NR A rare cause of pericardial constriction Int J Cardiol 2016 223497-499 10.1016/j.ijcard.2016.08.036
17	Fatima	Fatima U, Ilyas S, Zahid M, Mujtaba G, Riaz IB, Hashmi AT, et al A rare pericardial malignancy Circ Heart Fail 20169(10) e003282 10.1161/CIRCHEARTFAILURE.116.003282

18	Mensi	Mensi C, Romano A, Berti A, Dore R, Riboldi L A second case of pericardial mesothelioma mimicking systemic lupus erythematosus in the literature in over 30 years: a case report J Med Case Rep 2017 11(1) 85 10.1186/s13256-017-1237-z
19	Peralta-Amaro	Peralta-Amaro AL, Vázquez-Hernández A, Morales-Osorio G, Pecero-García E, Triana-González S, Manzo-Carballo F, et al A survivor woman after three years of cardiac tamponade J Cardiol Cases 2022 25(5) 259-26110.1016/j.jccase.2021.10.010
20	Ramachandran	Ramachandran R, Radhan P, Santosham R, Rajendiran S A rare case of primary malignant pericardial mesothelioma J Clin Imaging Sci 2014 4 47 10.4103/2156-7514.139737
21	Yan	Yan Y, Ye Y, Zhang F Aggressive primary pericardial mesothelioma Eur Heart J 2023 45(6) 49010.1093/eurheartj/ehad763
22	Kenchetty	Kenchetty PK, Balasundaram S, Rao K An uncommon aetiology for a common clinical problem: primary pericardial mesothelioma Natl Med J India 2022 35(1) 14-16
23	Kawakami	Kawakami N, Kawai M, Namkoong H, Sugiura H, Terai H, Ogata S, et al Cardiac tamponade due to primary malignant pericardial mesothelioma diagnosed with surgical pericardial resection J Cardiol Cases 2021 24(4) 149-15210.1016/j.jccase.2021.05.009
24	Massicotte	Massicotte G, Bernier M, Piché ME, Cantin L, Couture C Case report of aggressive primary pericardial mesothelioma presenting as a constrictive pericarditis Circ Cardiovasc Imaging 2019 12(3) e00862110.1161/CIRCIMAGING.118.008621
25	Gong	Gong J, Wu X, Wang J Case Report: A case of primary pericardial mesothelioma treated with multimodal combined therapy Front Cardiovasc Med 2024 11 1433668 10.3389/fcvm.2024.1433668
26	Raza	Raza SS, Ullah I, Awan SK, Nadeem MD, Bashir G Case Report: Can we differentiate mesothelioma from inflammatory pericardial constriction preoperatively? F1000Res 2020 9 513 10.12688/f1000research.23998.1

27	Wang	Wang D, Wang YH, Chu SC Case Report: Early diagnosis and bevacizumab-based chemotherapy for primary pericardial mesothelioma: a case with occupational asbestos exposure history Front Cardiovasc Med 2023 10 125737310.3389/fcvm.2023.1257373
28	Fujiwara	Fujiwara S, Kano Y Clinical response of primary malignant pericardial mesothelioma with peritoneal dissemination to nivolumab Intern Med 2024 63(4) 513-519 10.2169/internalmedicine.1707-23
29	Wu	Wu M, Li Z, Cai J, Li S, Zheng J Co-existing pericardial and pleural malignant mesothelioma responding well to nedaplatin and pemetrexed: a case report AME Case Rep 2023 7 32 10.21037/acr-22-102
30	Meijs	Meijs TA, Heidendael JF, Schurink B, Bugiani M, van Boven WJP, Boekholdt SM, et al Constrictive pericarditis caused by primary pericardial mesothelioma: a case series Circ Cardiovasc Imaging 2024 17(7) e01684710.1161/CIRCIMAGING.124.016847
31	Machii	Machii Y, Sezai A, Taoka M, Abe K, Shimokawa T Coronary artery stenosis caused by primary malignant pericardial mesothelioma in a 76-year-old man Tex Heart Inst J 2022 49(6) e207456 10.14503/THIJ-20-7456
32	Nicolini	Nicolini A, Perazzo A, Lanata S Desmoplastic malignant mesothelioma of the pericardium: description of a case and review of the literature Lung India 2011 28(3) 219-221 10.4103/0970-2113.83985
33	Liang	Liang L, Zhang X, Ke X, Yao S, Wu W Diagnosis and treatment of pericardial mesothelioma by mediastinal mass resection: a case report Ann Transl Med 2022 10(24) 1407 10.21037/atm-22-4719
34	Wang	Wang H, Mou Y, Zheng Z Diagnosis of primary pericardial mesothelioma by the combination of conventional echocardiography and contrast echocardiography Interact Cardiovasc Thorac Surg 2020 31(2) 274-27610.1093/icvts/ivaa086
35	Li	Li Q, Qin Z, Sun D, Hou C, Zhang S, Zhao Y Diffuse primary malignant pericardial mesothelioma with myocardial involvement: a comprehensive assessment

		using multimodel echocardiography Curr Probl Cardiol 2022 47(11) 10135610.1016/j.cpcardiol.2022.101356
36	Ashinuma	Ashinuma H, Shingyoji M, Yoshida Y, Itakura M, Ibi T, Sakaida T, et al Endobronchial ultrasound-guided transbronchial needle aspiration in a patient with pericardial mesothelioma Intern Med 2015 54(1) 43-4810.2169/internalmedicine.54.3272
37	Somani	Somani P, Sharma M, Patil A, Kumar A Primary pericardial mesothelioma diagnosed endoscopically Endoscopy 201648(Suppl 1) E45-E46 10.1055/s-0042-100470
38	Sivrikoz	Ak Sivrikoz İ, Önner H, Kasapoğlu Dündar E, Çavuşoğlu Y, Dernek S F-18 FDG PET/CT images of a rare primary cardiac tumour: primary pericardial mesothelioma Anatol J Cardiol 2016 16(8) 635-63610.14744/AnatolJCardiol.2016.7023
39	Uspenskiy	Uspenskiy V Primary pericardial mesothelioma presenting as constrictive pericarditis Interact Cardiovasc Thorac Surg2012 15(2) 301-303 10.1093/icvts/ivs194
40	Alwusaibie	Alwusaibie SA, Alsayigh JS, Alfaraj D, Alomair AM, Alsaeed SA Intermittent superior vena cava syndrome secondary to malignant pericardial mesothelioma Cureus 2020 12(12) e12107 10.7759/cureus.12107
41	Nady	Nady MA, Abdelrahman MY, Elgohary GM, Hafez MR, Abouelatta M, Abdelfattah K Localized isolated intrapericardial mesothelioma Turk Gogus Kalp Damar Cerrahisi Derg 2013 21(2) 536-539 10.5606/tgkdc.dergisi.2013.5740
42	Song	Song G, Bi W, Zhang X, Huang W, Zhou K, Ren W Localized primary malignant pericardial mesothelioma J Clin Ultrasound 2019 47(3) 178-181 10.1002/jcu.22673
43	Hai	Hai VA, Chau NTM, Hoanh HV, Tri HV, Pho DC, Nam NV, et al Long-term survival from multidisciplinary treatment of primary malignant pericardial mesothelioma: a case report Int J Surg Case Rep 2024 125 11061510.1016/j.ijscr.2024.110615

44	de Ceuninck	de Ceuninck M, Demedts I, Trenson S Malignant cardiac tamponade Acta Cardiol 2013 68(5) 505-50710.1080/AC.68.5.2994474
45	Sakuraba	Sakuraba M, Tatsumori T, Hirama M, Ogura K Malignant pericardial mesothelioma Eur J Cardiothorac Surg 2011 39(3)420 10.1016/j.ejcts.2010.07.012
46	Yıldırım	Yıldırım H, Metintaş M, Ak G Malignant pericardial mesothelioma following thoracic radiotherapy; dissemination from pericardium to pleura Tuberk Toraks 2010 58(3) 301-305
47	Steber	Steber C, Farris J, Hughes RT, Sayan M, Ohri N Malignant pericardial mesothelioma treated using volumetric modulated arc therapy with a simultaneous integrated boost Adv Radiat Oncol 2021 6(1) 100562 10.1016/j.adro.2020.09.002
48	Kim	Kim DY, Yoon YN, Hong GR, Ha JW, Shim CY Malignant pericardial mesothelioma: diagnostic clues in multimodality imaging Int J Cardiovasc Imaging 2020 36(8) 1385-1386 10.1007/s10554-020-01829-1
49	Savarrakhsh	Savarrakhsh A, Vakilpour A, Davani SZ, Farshidi H, Abdi A, Sadeghipour S, et al Malignant primary pericardial mesothelioma presenting as effusive constrictive pericarditis: a case report study J Cardiothorac Surg 2021 16(1) 29810.1186/s13019-021-01684-8
50	Morita	Morita S, Goto A, Sakatani T, Ota S, Murakawa T, Nakajima J, et al Multicystic mesothelioma of the pericardium Pathol Int 2011 61(5) 319-321 10.1111/j.1440-1827.2011.02654.x
51	Liu	Liu J, Wang Z, Yang Y, Sun Y, Wang S, Chen B, et al Multimodal diagnostic workup of primary pericardial mesothelioma: a case report Front Cardiovasc Med 2021 8 758988 10.3389/fcvm.2021.758988
52	Dung	Dung BT, Hien NT, Dinh NH, Truong NQ, Son PT, Van Bang N Multiple serous membrane effusion caused by primary pericardial mesothelioma Radiol Case Rep 2023 18(8) 2903-2906 10.1016/j.radcr.2023.06.013

53	Istomin	Istomin V, Blondheim DS, Meisel SR, Frimerman A, Atar S, Shachor J, et al Pericardial effusion due to primary malignant pericardial mesothelioma: a common finding but an uncommon cause Case Rep Med 2016 2016 481090110.1155/2016/4810901
54	Webb	Webb J, Yiu YW, Giastefani S, Carr-White G Primary pericardial mesothelioma presenting as constrictive pericarditisQJM 2016 109(9) 631-632 10.1093/qjmed/hcw060
55	Iliff	Iliff J, Bart NK, Ghaly S, Granger E, Holloway CJ Pericardial mesothelioma in a 35-year-old male with ulcerative colitisCirc Cardiovasc Imaging 2019 12(3) e008659 10.1161/CIRCIMAGING.118.008659
56	Barroso	Barroso AS, Leite S, Friões F, Marques J, Rocha S, Ribeiro VG Pericardial mesothelioma presenting as a suspected ST-elevation myocardial infarction Rev Port Cardiol 2016 35(4) 307.e1-5 10.1016/j.repc.2016.03.014
57	Berglund	Berglund F, Ali A, Sankar P, Seshadri N Pericardial mesothelioma presenting as constrictive pericarditisEchocardiography 2023 40(10) 1147-1150 10.1111/echo.15687
58	Shrestha	Shrestha B, Handa R, Poudel B, Hingorani R Pericardial mesothelioma presenting as constrictive pericarditis Cureus 202214(4) e24270 10.7759/cureus.24270
59	Laguna	Laguna G, Blanco M, Martínez G, Carrascal Y Pericardial pleomorphic mesothelioma and heart failure: a unique caseAnn Thorac Surg 2018 106(5) e321-e322 10.1016/j.athoracsur.2018.05.077
60	Chung	Chung SM, Kim HJ, Park SH, Lee SC, Park SW, Kim DK Positive response of a primary malignant pericardial mesothelioma Oncol Lett 2016 12(1) 213-216 10.3892/ol.2016.4598
61	Naeini	Naeini YB, Arcega R, Hirschowitz S, Rao N, Xu H Post-irradiation pericardial malignant mesothelioma with deletion of p16: a case report Cancer Biol Med 2018 15(1) 97-102 10.20892/j.issn.2095-3941.2017.0169
62	Bendek	Bendek M, Ferenc M, Freudenberg N Post-irradiation pericardial malignant mesothelioma: an autopsy case and review of the literature Cardiovasc Pathol 2010 19(6) 377-379 10.1016/j.carpath.2009.08.003

63	Schwartzenberg	Schwartzenberg S, Shapira Y, Rubachevski V, Sharony R Primary cardiac mesothelioma presenting with fulminant recurrent pericarditis: a case report Eur Heart J Case Rep 2023 7(3) ytad100 10.1093/ehjcr/ytad100
64	Patel	Patel J, Sheppard MN Primary malignant mesothelioma of the pericardium Cardiovasc Pathol 2011 20(2) 107-10910.1016/j.carpath.2010.01.005
65	Rosenbaum	Rosenbaum AN, Bois MC, Zhang X, Santivasi WL, Freeman WK Primary malignant pericardial mesothelioma: a clinical case series illustrating the necessity of a multidisciplinary approach JACC Case Rep 2019 1(2) 202-20710.1016/j.jaccas.2019.06.012
66	Horie	Horie M, Noguchi S, Tanaka W, Yoshihara H, Kawakami M, Suzuki M, et al Primary malignant pericardial mesothelioma mimicking pericardial metastasis from adenocarcinoma J Thorac Oncol 2010 5(12) 2012-201410.1097/JTO.0b013e3181f624da
67	Choi	Choi WS, Im MS, Kang JH, Kim JW, Park SC, Choi RK, et al Primary malignant pericardial mesothelioma presenting as acute pericarditis J Cardiovasc Ultrasound 2012 20(1) 57-59 10.4250/jcu.2012.20.1.57
68	Chen	Chen Y, Ren X, Duan X, Lu B Primary malignant pericardial mesothelioma presenting as constrictive pericarditis with an atypical imaging appearance: a case description Quant Imaging Med Surg 2023 13(3) 2013-2020 10.21037/qims-22-556
69	Matsuyama	Matsuyama S, Imazuru T, Uchiyama M, Ota H, Iida M, Shimokawa T Primary malignant pericardial mesothelioma presenting with cardiac tamponade Int J Surg Case Rep 2020 73 253-256 10.1016/j.ijscr.2020.07.054
70	Yan	Yan Y, Lv W, Luo Y, Hu J, Yang J Primary malignant pericardial mesothelioma with a survival of 2.5 years: a case reportTransl Cancer Res 2022 11(5) 1451-1456 10.21037/tcr-22-778
71	Oka	Oka N, Orita Y, Oshita C, Nakayama H, Teragawa H Primary malignant pericardial mesothelioma with difficult antemortem diagnosis: a case report World J Clin Cases 2022 10(33) 12380-12387 10.12998/wjcc.v10.i33.12380

72	Kurosawa	Kurosawa T, Sugino K, Isobe K, Hata Y, Fukasawa Y, Homma S Primary malignant pericardial mesothelioma with increased serum mesothelin diagnosed by surgical pericardial resection: a case report Mol Clin Oncol 2016 5(5) 553-55610.3892/mco.2016.1019
73	Gong	Gong W, Ye X, Shi K, Zhao Q Primary malignant pericardial mesothelioma-a rare cause of superior vena cava thrombosis and constrictive pericarditis J Thorac Dis 2014 6(12) E272-E275 10.3978/j.issn.2072-1439.2014.11.36
74	Ben Farhat	Ben Farhat S, Kammoun S, Frikha I, Hammami S, Ben Abdallah N, Frikha Z, et al Primary malignant pericardial mesothelioma: a case report Pan Afr Med J 2020 36 375 10.11604/pamj.2020.36.375.25336
75	Muta	Muta H, Sugita Y, Ohshima K, Otsubo H Primary malignant pericardial sarcomatoid mesothelioma: an autopsy reportPathol Int 2017 67(6) 311-315 10.1111/pin.12535
76	Tateishi	Tateishi K, Ikeda M, Yokoyama T, Sugio K, Yoneda R, Yoshida S, et al Primary malignant sarcomatoid mesothelioma in the pericardium Intern Med 2013 52(2) 249-253 10.2169/internalmedicine.52.8853
77	Reardon	Reardon KA, Reardon MA, Moskaluk CA, Grosh WW, Read PW Primary pericardial malignant mesothelioma and response to radiation therapy Rare Tumors 2010 2(3) e51 10.4081/rt.2010.e51
78	Seal	Seal S, Simon H Primary pericardial mesothelioma: a rare but serious consideration Cureus 2021 13(11) e1996610.7759/cureus.19966
79	Godar	Godar M, Liu J, Zhang P, Xia Y, Yuan Q Primary pericardial mesothelioma: a rare entity Case Rep Oncol Med 2013 2013283601 10.1155/2013/283601
80	Freitas	Freitas DMM, Ramos RL, Serpa Pinto L, Ribeiro R Primary pericardial mesothelioma and asbestos exposure: a rare fatal disease BMJ Case Rep 2017 2017 bcr-2017-219949 10.1136/bcr-2017-219949
81	Zhang	Zhang J, Liu D, Zhang D, Guo K, Yang X Primary pericardial mesothelioma complicated by pericardial calcificationBMC Cardiovasc Disord 2023 23(1) 125 10.1186/s12872-023-03142-w

82	Kayatta	Kayatta MO, Dineen SP, Sica G, Puskas JD, Pickens A Primary pericardial mesothelioma in a 19-year-old presenting as pericarditis Ann Thorac Surg 2013 96(2) 680-681 10.1016/j.athoracsur.2012.12.043
83	Kowalczuk-Wieteska	Kowalczuk-Wieteska A, Filipiak K, Miszalski-Jamka K, Nozynski J, Zembala M Primary pericardial mesothelioma in a 48-year-old patient Pol Arch Intern Med 2017 127(11) 792-793 10.20452/pamw.4155
84	Rizzardi	Rizzardi C, Barresi E, Brollo A, Cassetti P, Schneider M, Melato M Primary pericardial mesothelioma in an asbestos-exposed patient with previous heart surgery Anticancer Res 2010 30(4) 1323-1326
85	Yamamoto	Yamamoto T, Takagi H, Kawai N, Umemoto T Primary pericardial mesothelioma presenting as constrictive pericarditisEur Heart J Cardiovasc Imaging 2019 20(5) 602 10.1093/ehjci/jey224
86	Raeside	Raeside MC, Gormly K, Neuhaus SJ, Kotasek D, James C Primary pericardial mesothelioma presenting as multiple pericardial masses on CT: a case report BJR Case Rep 2016 2(2) 20150295 10.1259/bjrcr.20150295
87	Sardar	Sardar MR, Kuntz C, Patel T, Saeed W, Gnall E, Imaizumi S, et al Primary pericardial mesothelioma: unique case and literature review Tex Heart Inst J 2012 39(2) 261-264
88	Tajjiou	Tajjiou M, Wild W, Sayed N, Flauaus A, Divo M, Schwarzbach M Primary pericardial mesothelioma, which was veiled by a pleural empyema: a case report and review Case Rep Surg 2019 2019 2896810 10.1155/2019/2896810
89	Arponen	Arponen O, Salo V, Lönnberg A, Vaalavirta L, Koivu H, Nyandoto P Primary pericardial mesothelioma treated with an immune checkpoint inhibitor as second-line treatment Acta Oncol 2021 60(5) 687-691 10.1080/0284186X.2021.1887515
90	Belli	Belli E, Landolfo K Primary pericardial mesothelioma: a rare cause of constrictive pericarditis Asian Cardiovasc Thorac Ann 2015 23(5) 599-600 10.1177/0218492313517384
91	Terada	Terada T Primary sarcomatoid malignant mesothelioma of the pericardium Med Oncol 2012 29(3) 1345-134610.1007/s12032-011-9854-7

92	Jiang	Jiang D, Kong M, Li J, Qian J Primary sarcomatoid malignant pericardial mesothelioma Intern Med 2013 52(1) 157-15810.2169/internalmedicine.52.8934
93	Rodríguez	Rodríguez M, Mallidi HR, da Silva A, Bueno R Recurrence of pericardial mesothelioma affecting the myocardium after pericardial resection Ann Thorac Surg 2018 106(5) e243-e245 10.1016/j.athoracsur.2018.04.057
94	Kashinath	Kashinath S, Vuyyala S, Baig B, Verghese B Recurrent pericardial effusions and pleural effusion: what are we missing? A rare case of primary malignant pericardial mesothelioma Chest 2018 154(4) 476A 10.1016/j.chest.2018.08.433
95	Saisho	Saisho C, Ishii H, Edakuni N, Imamura Y, Tokito T, Kinoshita T, et al Sarcomatoid type primary pericardial mesothelioma with a long-term survival after the onset of cardiac tamponade Intern Med 2016 55(21) 3161-316410.2169/internalmedicine.55.6300
96	Isoda	Isoda R, Yamane H, Nezuo S, Nomura T, Nii H, Ishii M, et al Successful palliation for an aged patient with primary pericardial mesothelioma World J Surg Oncol 2015 13 273 10.1186/s12957-015-0692-5
97	Martínez-Girón	Martínez-Girón R, Pantanowitz L, Martínez-Torre S, Pantanowitz J Sudden cardiac death due to primary malignant pericardial mesothelioma: brief report and literature review Respir Med Case Rep 2019 26 185-18810.1016/j.rmcr.2019.01.011
98	Apicella	Apicella G, Boulemden A, Citarella A, Sushma R, Szafranek A Surgical treatment of a primary malignant pericardial mesothelioma: case report Acta Chir Belg 2022 122(1) 48-50 10.1080/00015458.2020.1736869
99	Vavalle	Vavalle J, Bashore TM, Klem I Surprising finding of a primary pericardial mesothelioma Int J Cardiovasc Imaging 201026(6) 625-627 10.1007/s10554-010-9607-5
100	Shikano	Shikano K, Hirano S, Hiroishi T, Morichika D, Miura A, Fujii S, et al The antemortem diagnosis of primary malignant pericardial mesothelioma: a multidisciplinary evaluation Intern Med 2018 57(17) 2559-256210.2169/internalmedicine.0355-17

101	Utrera-Costero	Utrera-Costero A, Bernal-Vergara JC, Olivan-Sasot P, Yepes-Agudelo AM, Ruiz-Llorca C, Vera-Pinto VJ, et al The value of 18F-FDG PET/CT in two cases of primary pericardial mesothelioma J Nucl Cardiol 2022 29(6) 3604-360710.1007/s12350-021-02690-y
102	Fujita	Fujita K, Hata M, Sezai A, Minami K Three-year survival after surgery for primary malignant pericardial mesothelioma: report of a case Surg Today 2014 44(5) 948-951 10.1007/s00595-013-0511-y
103	Chen	Chen HC, Chang S, Hsiung MC, Kwang WK, Tsao TP, Yang YN Timely diagnosis of primary pericardial mesotheliomaEchocardiography 2020 37(11) 1877-1879 10.1111/echo.14888
104	Oc	Oc M, Aydin U, Sarioglu T, Yilik L, Gurbuz A Unexpected malignant pericardial mesothelioma presenting as pericardial constriction Bratisl Lek Listy 2012 113(10) 620-621 10.4149/BLL_2012_140
105	Günday	Günday M, Erinanc H, Geredeli C Unusual region for pericardial malignant mesothelioma: cutaneous manifestation in a Turkish woman Rare Tumors 2013 5(3) e41 10.4081/rt.2013.e41
106	Zhang	Zhang Y, Pang M Unveiling the mystery: a rare case of localized malignant pericardial mesothelioma: case report Front Oncol 2024 14 1342748 10.3389/fonc.2024.1342748
107	Pölzl	Pölzl L, Hirsch J, Mayr A, Müller S, Grimm M, Gollmann-Tepeköylü C When cardiac surgery comes to its limits: a case report of pericardial mesothelioma invading the myocardium Eur Heart J Case Rep 2021 5(7) ytab23710.1093/ehjcr/ytab237
108	Makarawate	Makarawate P, Chaosuwannakit N, Chindaprasirt J, Sawanyawisuth K Malignant mesothelioma of the pericardium: a report of two different presentations Case Rep Oncol Med 2013 2013 356901 10.1155/2013/356901
109	Hirnle	Hirnle G, Kapałka M, Krawiec M, Hrapkowicz T Pericardial mesothelioma mimicking mediastinal lymphoma and systemic rheumatic disease: a case report Front Oncol 2025 14 1481373 10.3389/fonc.2024.1481373

110	Hatta	Hatta SSBWM, Mirza AR, Sunni N, Bashir A Heart-breaking tumours: a case series of malignant pericardial effusion Eur Heart J Case Rep 2025 9(2) ytaf009 10.1093/ehjcr/ytaf009
111	Luebbe	Luebbe ST, Youmans Q, Provias T, Hoit B, Gelfand EV, Kim J, et al Primary pericardial mesothelioma causing constrictive pericarditis JACC Case Rep 2025 30(8) 103204 10.1016/j.jaccas.2025.103204
112	Rathore	Rathore H A rare primary pericardial mesothelioma on an FDG PET/CT scan Indian J Nucl Med 2024 39(4) 313-31510.4103/ijnm.ijnm_43_24
