# Supplementary material for: Prognostic Factors and Survival in Pericardial Mesothelioma: A Systematic Review and Quantitative Analysis
Source: Cancers (Basel). 2026 Aug 11;18(16):2583. doi: 10.3390/cancers18162583 (PMC13511537; doi:10.3390/cancers18162583)
Supplement: Supplementary file 1 [file cancers-18-02583-s001.zip › Supplementary Tables - logistic regression S7 and S8.pdf]

Supplementary Table S7. Logistic regression analysis for survival >12 months (from symptom onset).

Factor	OR	LCI	UCI	p-value
Sex	0,838207	0,313420	2,241688	0,725108
Azbest exposure	1,517045	0,380475	6,048824	0,554796
Cardiovascular comorbidities	0,442577	0,094676	2,068900	0,300211
Past tumors	0,314815	0,039050	2,537989	0,277769
Other comorbidities	0,945455	0,284727	3,139442	0,927015
Localised mesothelioma	1,027295	0,357306	2,953591	0,960140
Diffused mesothelioma	1,386986	0,422792	4,550060	0,589403
Constrictive pericarditis	0,758929	0,251571	2,289504	0,624389
Presence of effusion	2,986486	0,645529	13,81673	0,161534
Cytology test	1,358025	0,508182	3,629077	0,541719
Cytology result	1,866667	0,672610	5,180486	0,230739
Misdiagnosis	3,366379	1,228608	9,223860	0,018260
Treated as chronic idiopathic pericarditis	2,193878	0,682997	7,047032	0,186969
Treatment as for other specific pericarditis type	0,915441	0,239952	3,492494	0,897101
Diagnosis confirmed by surgical biopsy	1,019608	0,355146	2,927251	0,971213
Diagnosis confirmed by percutaneous biopsy	1,066667	0,319072	3,565898	0,916526
Thoracic biopsy	1,933131	0,703034	5,315527	0,201526

PET	2,466899	0,895121	6,798621	0,080854
MRI	2,850000	1,037264	7,830700	0,042264
CT scan	2,684211	0,578125	12,46267	0,207505
Local metastases	2,928571	0,869849	9,859789	0,082769
Distal metastases	0,666667	0,139447	3,187190	0,611508
Surgery	0,798113	0,298811	2,131733	0,652796
MRI	2,850000	1,037264	7,830700	0,042264
Chemotherapy	0,300505	0,108377	0,833232	0,017010
Immunotherapy	2,823529	0,479020	16,64297	0,251466
Radiotherapy	1,352941	0,264130	6,930116	0,716850
Continuous factors				
age [years]	1,037718	1,006639	1,069756	0,017010
Pericardial mesothelioma thickening [mm]	0,775994	0,311812	1,931182	0,585627
Tumor volume [cm3]	0,998636	0,996896	1,000379	0,125115
SUVmax	1,044239	0,854746	1,275741	0,671776
Effusion [mm]	1,557093	1,047267	2,315111	0,028657
Drainage [ml]	0,999852	0,998780	1,000925	0,787336
Diagnostics time [months]	0,954539	0,915234	0,995532	0,030105

Supplementary Table S8 Logistic regression analysis for survival >12 months (from diagnosis).

Factor	OR	LCI	UCI	p-value

Sex	0,622024	0,253638	1,525456	0,299588
Azbest exposure	1,447619	0,412195	5,083998	0,563824
Cardiovascular comorbidities	0,979592	0,291277	3,294462	0,973419
Past tumors	0,233974	0,029068	1,883318	0,172235
Other comorbidities	0,450000	0,121918	1,660954	0,230745
Localised mesothelioma	1,165179	0,458383	2,961803	0,748081
Diffused mesothelioma	0,775794	0,295118	2,039375	0,606683
Constrictive pericarditis	0,355556	0,111734	1,131431	0,079966
Presence of effusion	2,838710	0,777489	10,36449	0,114324
Cytology test	1,971698	0,714165	5,443549	0,190110
Cytology result	7,125000	1,702934	29,81069	0,007167
Misdiagnosis	0,482972	0,175111	1,332078	0,159719
Treated as chronic idiopathic pericarditis	0,901587	0,270034	3,010213	0,866253
Treatment as for other specific pericarditis type	1,285714	0,413278	3,999874	0,664289
Diagnosis confirmed by surgical biopsy	0,730469	0,276925	1,926822	0,525663
Diagnosis confirmed by percutaneous biopsy	0,944444	0,311507	2,863422	0,919549
Thoracic biopsy	0,553191	0,223610	1,368547	0,200167
PET	1,305556	0,535007	3,185895	0,558019
MRI	1,004630	0,372523	2,709312	0,992719

CT scan	3,484848	0,755303	16,07853	0,109545
Local metastases	0,256944	0,031739	2,080085	0,202822
Distal metastases	2,701754	0,856725	8,520209	0,089871
Surgery	1,936111	0,755652	4,960654	0,168725
Chemotherapy	3,318519	1,310274	8,404783	0,011409
Immunotherapy	0,675000	0,075181	6,060412	0,725600
Radiotherapy	0,356481	0,042953	2,958587	0,339412
Continuous factors				
age [years]	0,980905	0,952989	1,009639	0,190618
Pericardial mesothelioma thickening [mm]	0,592189	0,238610	1,469715	0,258608
Tumor volume [cm3]	1,000888	0,998646	1,003135	0,437831
SUVmax	0,796390	0,576193	1,100737	0,167982
Effusion [mm]	1,040940	0,891963	1,214798	0,610642
Drainage [ml]	0,999832	0,998815	1,000851	0,747010
Diagnostics time [months]	0,983274	0,946095	1,021913	0,391055
